# ChIP-AP: an integrated analysis pipeline for unbiased ChIP-seq analysis

**DOI:** 10.1093/bib/bbab537

**Published:** 2021-12-30

**Authors:** Jeremiah Suryatenggara, Kol Jia Yong, Danielle E Tenen, Daniel G Tenen, Mahmoud A Bassal

**Affiliations:** Cancer Science Institute of Singapore, National University of Singapore, Singapore, 117599, Singapore; Cancer Science Institute of Singapore, National University of Singapore, Singapore, 117599, Singapore; Department of Biochemistry, Yong Loo Lin School of Medicine, National University of Singapore, Singapore, 117597, Singapore; Broad Institute of MIT and Harvard, Boston, 02142, USA; Cancer Science Institute of Singapore, National University of Singapore, Singapore, 117599, Singapore; Harvard Stem Cell Institute, Boston, 02138, USA; Cancer Science Institute of Singapore, National University of Singapore, Singapore, 117599, Singapore; Harvard Stem Cell Institute, Boston, 02138, USA

**Keywords:** ChIP-seq, integrated analysis pipeline, multiple peak callers, automated analysis pipeline, transcription factor binding, histone mark

## Abstract

Chromatin immunoprecipitation coupled with sequencing (ChIP-seq) is a technique used to identify protein–DNA interaction sites through antibody pull-down, sequencing and analysis; with enrichment ‘peak’ calling being the most critical analytical step. Benchmarking studies have consistently shown that peak callers have distinct selectivity and specificity characteristics that are not additive and seldom completely overlap in many scenarios, even after parameter optimization. We therefore developed ChIP-AP, an integrated ChIP-seq analysis pipeline utilizing four independent peak callers, which seamlessly processes raw sequencing files to final result. This approach enables (1) better gauging of peak confidence through detection by multiple algorithms, and (2) more thoroughly surveys the binding landscape by capturing peaks not detected by individual callers. Final analysis results are then integrated into a single output table, enabling users to explore their data by applying selectivity and sensitivity thresholds that best address their biological questions, without needing any additional reprocessing. ChIP-AP therefore presents investigators with a more comprehensive coverage of the binding landscape without requiring additional wet-lab observations.

## Introduction

Chromatin immunoprecipitation coupled with sequencing (ChIP-seq) is used to identify DNA-binding location and recognition motifs of DNA-interacting proteins such as transcription factors [1, 2], histone-modifiers [3] and novel DNA-binding proteins. Since ChIP-seq’s inception [2, 4, 5], its computational analysis has been a complex, multi-step, command-line driven process requiring knowledge and experience in computing and programming [6]. For each step within an analysis, many analogous programs have been developed complicating decisions of which programs to use in conjunction with one-another. Additional complications arise when attempting to run these tools sequentially as input/output inconsistencies necessitate manual intervention, format conversions, or even, data filtering between steps. Therefore, two different analysis methodologies, even if appearing superficially similar, will most certainly report different results, leading to potentially conflicting conclusions [7, 8]. For these reasons, it is difficult for researchers inexperienced with ChIP-seq computational analysis to process their own data without dedicating a significant amount of time to learn coding, research the required tools and then determine how to best integrate everything together to produce valid results.

Of all the programs required for a ChIP-seq analysis, the choice of peak caller is the most critical [7]. In 2016, Steinhauser *et al.* [8] compared 20 peak callers and reported poor peak agreement between all profiled callers. This and other studies show that peak callers have distinct selectivity and specificity characteristics that are often not additive and seldom completely overlap in many scenarios even after optimizing configuration parameters [7–11]. Consequently, such differing operating characteristics results in inconsistency across reported regions of enrichment (and associated genes) with downstream effects on functional analysis that can result in differing and potentially conflicting results from the same dataset. The performance of a peak caller is also drastically affected by the distribution and quality of the sequenced reads [8–12]. An individual peak caller can show superior performance in certain datasets, but not others. Therefore, relying on a one-caller-fits-all approach when analyzing ChIP-seq datasets with different DNA-binding proteins, immunoprecipitation and library preparation protocols, is objectively not the most robust approach to extract the most reliable or comprehensive results from a particular dataset.

We therefore rationalized that to improve the reliability and comprehensiveness of ChIP-seq analyses, one should improve the consistency, confidence and comprehensiveness of the peak detection step without requiring additional wet-lab observations. To address this, we developed an integrated ChIP-seq Analysis Pipeline (ChIP-AP), which caters to all user proficiencies by being accessible through either graphical interface, or command-line. ChIP-AP seamlessly performs all analysis steps processing raw sequencing files to final result, utilizes four peak callers [13–17] and generates a single integrated output file. By integrating the results of multiple peak callers, users can focus on addressing specific questions. For example, the biological question may be to identify the best consensus binding site for a certain transcription factor. In other cases, the aim is to identify as many possible gene targets. No individual peak caller can address all the different questions in any given dataset. ChIP-AP enables the user to investigate peak subsets acquired by different peak calling algorithms to obtain the best possible answer without requiring data re-analysis. ChIP-AP can therefore become an effective tool by providing substantial improvements to peak capturing and analysis confidence, aiding in avoiding the frequent cases where using only one peak caller leads to the conclusion that the experiment produced no useful results.

ChIP-AP is available through GitHub (https://github.com/JSuryatenggara/ChIP-AP). Alternatively, for users without compatible hardware, ChIP-AP has been incorporated into the CSI NGS Web Portal [18] for easy access (https://csibioinfo.nus.edu.sg/csingsportal).

## Materials and methods

### ChIP-AP constituent programs

ChIP-AP is an analysis pipeline that seamlessly integrates multiple command line tools into a single workflow. At time of publication (ChIP-AP version 5.0), these tools include FastQC [19], Clumpify and BBDuk from the BBMap Suite [20], Trimmomatic [21], BWA [22], Samtools [23], deepTools [24], MACS2 [25], GEM [16], SICER2 [26], HOMER [15], Genrich [13], IDR [27] and the MEME-Suite [28]. However, it is best to refer to the GitHub repository for the latest citation list that would include any additional tools incorporated into ChIP-AP since publication.

### Tissue culture/SNU-398 cell culture

The SNU-398 cell line was obtained from the American Type Culture Collection (ATCC). The cells were maintained in RPMI medium supplemented with 10% fetal bovine serum (FBS) at 37°C in a humidified atmosphere of 5% CO_2_ as recommended by ATCC.

### SNU-398 SALL4 ChIP-seq preparation and sequencing

20 million SNU-398 cells were cross-linked with 1% formaldehyde for 10 min at room temperature. The reaction was terminated by adding 2 M glycine to a final concentration of 125 mM. Cells were then washed with 1× PBS and resuspended in 1 ml of cell lysis buffer (20 mM Tris pH 8.0, 85 mM KCl, 0.5% nonidet P-40, protease inhibitor). After 10 min of incubation on ice, cells were spun down and the cell pellet resuspended in another 1 ml of cell lysis buffer. After another 5 min of incubation on ice, cells were spun down and cell pellet resuspended in 1 ml of nuclear lysis buffer (10 mM Tris-HCl pH 7.5, 1% nonidet P-40, 0.5% sodium deoxycholate, 0.1% SDS, protease inhibitor). After 10 min of incubation on ice, chromatin was sheared to 500 bp. Antibody-protein A/G Dynabead conjugate was prepared by adding 0.75 μg of SALL4 rabbit monoclonal antibody (Cell Signaling Technology #8459) to pre-washed 50 μl of protein A/G Dynabeads (Life Technologies) with 1 h incubation at 4°C with rotation. Sheared chromatin was then added to the antibody–protein A/G conjugate and incubated overnight at 4°C with rotation. After overnight incubation, the beads were washed sequentially with the following buffers: twice with RIPA/500 mM NaCl buffer (0.1% deoxycholate, 0.1% SDS, 1% Triton X-100, 500 mM NaCl, 1 mM EDTA, 20 mM Tris-HCl pH 8.1), twice with LiCl buffer (0.25 M LiCl, 1% nonidet P-40, 1% sodium deoxycholate, 1 mM EDTA, 10 mM Tris-HCl pH 8.1), twice with TE buffer (10 mM Tris-HCl pH 8.0, 1 mM EDTA pH 8.0). Protein complexes were reverse cross-linked with 50 μl of ChIP Elution Buffer (10 mM Tris-HCl pH 8.0, 5 mM EDTA, 300 mM NaCl, 0.1% SDS) and 8 μl of Reverse Crosslink Mix (250 mM Tris-HCl pH 6.5, 1.25 M NaCl, 62.5 mM EDTA, 5 mg/ml proteinase K, 62.5 μg/ml RNase A) at 65°C for 5 h. Reverse cross-linked DNA was cleaned up using SPRI beads (Beckman Coulter) and eluted in 10 mM Tris-HCl pH 8.0. To generate libraries for deep sequencing, the eluted DNA was end-repaired using End-It DNA End-Repair Kit (Epicenter #ER0720) and A-tailing was then carried using Klenow (3′–5′ exo-) enzyme (New England Biolabs). Illumina sequencing adaptors were ligated to the DNA fragments and adaptor-ligated DNA fragments were enriched with 14 cycles of PCR. DNA libraries were gel purified and analyzed on Bioanalyzer (Agilent) for their size distribution. Libraries were sequenced on Illumina HiSeq 2500 sequencer with single-end 35 bp settings.

**Table:** The SALL4 ChIP-seq was processed with ChIP-AP (v5.1) using hg38. Below is the settings table (ST) used.

Program	Argument
fastqc1	-q
clumpify	dedupe spany addcount qout=33
bbduk	ktrim=l hdist=2
trimmomatic	LEADING:20 SLIDINGWINDOW:4:20 TRAILING:20 MINLEN:20
fastqc2	-q
bwa_mem	
samtools_view	
plotfingerprint	-bs 50 --centerReads –ignoreDuplicates
fastqcs3	-q
macs2_callpeak	
gem	-Xmx30G --k_min 8 --k_max 12
sicer2	
homer_findPeaks	
genrich	--adjustp –v
homer_mergePeaks	
homer_annotatePeaks	
fold_change_calculator	--normfactor uniquely_mapped
homer_findMotifsGenome	-size given -mask
meme-chip	-neg background.fa -meme-nmotifs 25 target.fa

### SNU-398 SALL4 ChIP-seq analysis and comparisons

For this analysis, the union peak set was utilized. The fingerprint plot (Figure 4A) was generated as part of the ChIP-AP run with the flags outlined in the ST. The upset plot (Figure 4B) was generated by taking the ‘venn.txt’ data from the ChIP-AP run output (Folder 21_peaks_merging) and plotting in R [29] (v4.0.3) with the UpSetR [30] (v1.4.0) package. Irreproducible discovery rate (IDR) distribution plots were generated using Prism (v9.2.0) using the values reported in the final integrated ChIP-AP output file.

The Cut&Run dataset was processed as outlined previously [31] (using CnRAP) and is available from GEO, Accession GSE136332. To overlap the three Cut&Run replicates, HOMER’s mergePeaks was used with flags ‘-d 1500.’ Next, the Cut&Run peaks identified in at least two replicates with at least a 4-fold change over IgG were combined and compared to the SALL4 ChIP-seq union peak set using HOMER’s mergePeaks with flag ‘-d 2000’. This provided the list of overlapping regions, the number of which was plotted in R (v4.0.3) with VennDiagram [32] (v1.6.20). Different sized merge windows were used for the overlapping and comparison owing to the different peak shape characteristics of Cut&Run and ChIP-seq peaks.

For the directed motif search within the SALL4 ChIP-seq union peak set, HOMER’s findMotifsGenome was used with flags ‘-find sall4_weighted_motif.motif.’ *De novo* motif searching is incorporated into ChIP-AP and was performed using HOMER and the MEME-Suite with parameters shown in the ST. Motif logo files were generated using R (v4.0.3) and seqLogo [33] (v1.52.0).

The Gene Ontology (GO) analysis of the SALL4 ChIP-seq was performed as part of the ChIP-AP run using the flag ‘-goann’ which utilizes HOMER. To compare with the processed SALL4 knock-down published results [31], we started from Supplementary Tables 4 and 5, available online at http://bib.oxfordjournals.org/, from the publication. Next, we overlayed the reported gene names from the SALL4 ChIP-seq union peaks to those gene lists to determine overlapping gene names.

**Table:** A number of ENCODE datasets were downloaded and utilized; experiment IDs are listed below. Data were downloaded from ENCODE March 2021.

Cell line	Transcription factor	ChIP experiment ID’s	Control experiment ID’s
GM12878	MAX	ENCFF000VXY	ENCFF000VWF ENCFF000VWH
		ENCFF000VYA	
	SPI1	ENCFF000OBS	
		ENCFF000OB	
H1-hESC	CTCF (Bernstein)	ENCFF000AVU	ENCFF036EGF ENCFF191QXK
		ENCFF000AVS	
	CTCF (Myers)	ENCFF000ONR	ENCFF000OSP
		ENCFF000OOF	
	REST (Bernstein)	ENCFF794IGW	ENCFF036EGF ENCFF191QXK
		ENCFF079NVQ	
	REST (Myers)	ENCFF000OQQ	ENCFF000OSP
		ENCFF000OQY	
	H3K79me2 (Bernstein)	ENCFF000AYD	ENCFF036EGF ENCFF191QXK
		ENCFF000AYF	
	H3K79me2 (Ren)	ENCFF580OHZ	ENCFF835IOE ENCFF094GYG
		ENCFF519ZRJ	
HepG2	ZBTB33	ENCFF000PSP	ENCFF000POC ENCFF000POH
		ENCFF000PSW	
	CEBPB	ENCFF000XQM	
		ENCFF000XQN	
	JUND (Myers)	ENCFF000PKK	
		ENCFF000PKR	
	JUND (Snyder)	ENCFF000XTQ ENCFF000XTR	ENCFF002ECQ ENCFF002ECU
K562	MAFF	ENCFF000YSQ	ENCFF002EFF ENCFF002EFD
		ENCFF000YSS	
	JUN	ENCFF000YJJ	
		ENCFF000YJL	
	GATA1	ENCFF000YND	
		ENCFF000YNF	
	MYC (Iyer)	ENCFF000RWD	ENCFF000RWS
		ENCFF000RWE	
		ENCFF000RWG	
	MYC (Snyder)	ENCFF000YKO	ENCFF002ECS ENCFF002ECW
		ENCFF000YKR	
	YY1 (Farnham)	ENCFF000ZEK	ENCFF000VEK
		ENCFF000ZEJ	
	YY1 (Myers)	ENCFF000QKF	ENCFF000QET ENCFF000QEU
		ENCFF000QKI	
	H3K4me1 (Bernstein)	ENCFF000BXX ENCFF000BYG	ENCFF000BWK ENCFF994FIB ENCFF283HQV ENCFF156ECZ ENCFF561WFK
	H3K4me1 (Farnham)	ENCFF000VDV	ENCFF000VEK
		ENCFF000VDU	
	H3K27me3 (Bernstein)	ENCFF000BXP	ENCFF000BWK ENCFF994FIB ENCFF283HQV ENCFF156ECZ ENCFF561WFK
		ENCFF000BXN	



	H3K27me3 (Farnham)	ENCFF000VDN	ENCFF000VEK
		ENCFF000VDP	
	H3K36me3 (Bernstein)	ENCFF000BXR	ENCFF000BWK ENCFF994FIB ENCFF283HQV ENCFF156ECZ ENCFF561WFK
		ENCFF000BXO	



	H3K36me3 (Stamatoyannopoulos)	ENCFF001FWV	ENCFF001HTT
		ENCFF001FWW	
	MEIS2	R1: ENCFF002EIU	R1: ENCFF002EFF ENCFF002EFD R2: ENCFF002EFH ENCFF002EFA
		ENCFF002EIW	
		R2: ENCFF002EIV	
		ENCFF002EIX	
	RUNX1	R1: ENCFF002DOZ		
		ENCFF002EGD		
		R2: ENCFF002EGE		
		ENCFF002DPH		
	ATF4	R1: ENCFF081USS		
		ENCFF565KLI		
		R2: ENCFF069VNL		
		ENCFF682IGK		
MCF7	H3K9me3 (Bernstein)	ENCFF656BIN	ENCFF318ZNB ENCFF640MKX ENCFF222VRH ENCFF595BTS
		ENCFF517BLK	
		ENCFF600JOS	

	H3K9me3 (Farnham)	ENCFF000VFE	ENCFF000VHL ENCFF000VHM
		ENCFF000VFJ	
		ENCFF000VFG	

**Table:** All ENCODE datasets were processed with ChIP-AP (v5.1), using hg38 with the following ST.

Program	Argument
fastqc1	-q
clumpify	dedupe spany addcount qout=33 fixjunk
bbduk	ktrim=l hdist=2
trimmomatic	LEADING:20 SLIDINGWINDOW:4:20 TRAILING:20 MINLEN:20
fastqc2	-q
bwa_mem	
samtools_view	
plotfingerprint	-bs 50 --centerReads –ignoreDuplicates
fastqcs3	-q
macs2_callpeak	
gem	-Xmx30G --k_min 8 --k_max 12
sicer2	
homer_findPeaks	
genrich	--adjustp -v
homer_mergePeaks	
homer_annotatePeaks	
fold_change_calculator	--normfactor uniquely_mapped
homer_findMotifsGenome	-size given -mask
meme-chip	-meme-nmotifs 25

### Encode datasets utilized and processing

The IDR calculations for all peaks are integrated into a ChIP-AP run. Once all individual peak callers have run, the union peak set is generated by merging results from all peak callers using HOMER’s mergePeaks with parameters as shown in the ST. The union peakset set is then ranked by (1) number of detecting peak callers, and (2) fold change of sample signal over input. As the IDR suite calculates peak reproducibility rate between two replicates (i.e. peak sets) only, we chose as ‘replicates’ each individual peak caller set and the union peak set. This allowed us to calculate reproducibility of all peaks in the full (union) peaks list based in their detectability by different individual peak callers and rank them accordingly. We then copy the individual peak IDR information to the union peak set giving us four −log_10_ IDR values for every peak. These four −log_10_ IDR values are then summed and converted into a final IDR value per peak.

For each transcription factor, the corresponding JASPAR binding motif was downloaded from MethMotif [34] in MEME format and converted to HOMER’s motif format. For the directed motif searches, HOMER’s findMotifsGenome was utilized with flags ‘hg38 -find binding_motif.motif.’ *De novo* motif searching is incorporated into ChIP-AP and was performed using HOMER and the MEME-Suite with parameters shown in the ST. Motif logo files were generated using R (v4.0.3) and seqLogo (v1.52.0). The GO results were generated as part of HOMER’s annotatePeaks function for the required peak sets. HOMER annotatePeaks was utilized with a known motif provided with the ‘-m’ flag to include the distances from all starting coordinate motif instances in each peak to their respective peak starting coordinate. A custom python script was utilized to extract the distances from every peak’s weighted peak center to the midpoint coordinate of the motif instance closest to the weighted peak center. The weighted peak center for transcription factor peaks is calculated using a separate custom script (which is incorporated into a ChIP-AP analysis), and is independent of any peak center defined by any individual peak caller utilized. The density plots representing this data were generated using R and ggplot2. Peak-Motif percentages were plotted using Graphpad Prism v9.2.0.

## ChIP-AP functionality and characteristics

### Design and modularity

**Figure 1:**
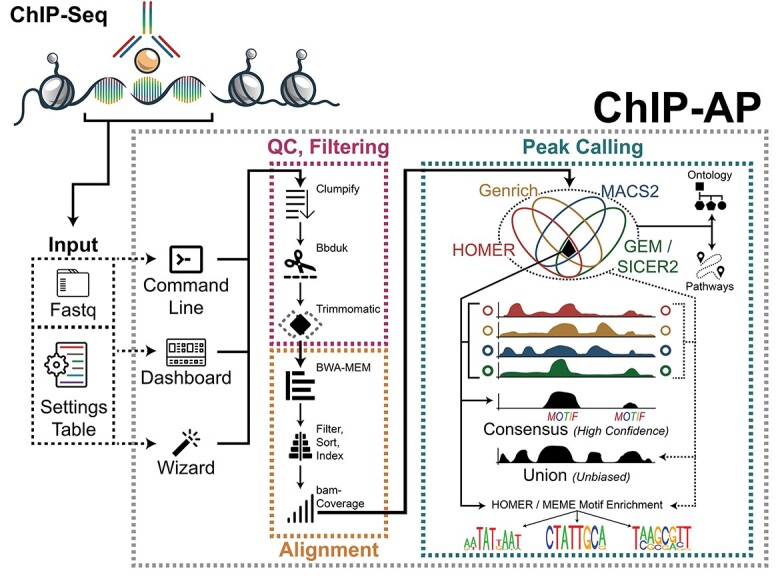
ChIP-AP functionality and characteristics. An overview of ChIP-AP’s structure. For input, ChIP-AP requires ST [be it customized for a specific analysis or the default (DST)] in conjunction with the location of the raw sequencing FASTQ files. Data can be entered through the wizard (Supplementary Figure 1A available online at http://bib.oxfordjournals.org/) or the dashboard (Supplementary Figure 1B available online at http://bib.oxfordjournals.org/) or the command-line. Once all data are entered, ChIP-AP constructs a folder hierarchy, each with sub-scripts that have defined input/output characteristics performing a singular task. The core stages of analysis include QC & Filtering, Alignment and Peak Calling. For output, ChIP-AP generates an integrated results table that can be viewed in any spreadsheet program like Excel, and can select the union peak set, the consensus peak set or any sub-set in-between as required. Integrated into the output file is all the peak callers that called said peak as well as the peak’s IDR. Optionally, users can run pathway and GO analysis which (if run) have their results merged into the main text output file as well. Users can also optionally run motif enrichment analysis using HOMER and/or MEME-ChIP, which will report results in separate output folders for viewing.

ChIP-AP’s design is inspired by, and copies modularity concepts from ‘object-oriented’ paradigms but is not ‘object-oriented’ in the truest sense. To run ChIP-AP, the location of the raw FASTQ files and a ST are passed through the command line or graphical interfaces (Figure 1**—**Input). ChIP-AP then constructs a structured folder hierarchy and generates analysis sub-scripts for each stage of analysis. Each sub-script script is, in essence, an ‘object’ with defined inputs and outputs. ChIP-AP therefore provides a platform wherein individual stages can be modularly swapped with equivalent steps without changes to the flow of analysis provided users are mindful of adjoining objects requisite parameters. This compartmentalization enables exponential customization of ChIP-AP for users.

### Peak caller selection

Improving the confidence and comprehensiveness of peak detection is the key focus of ChIP-AP. For our selection, a peak caller needed to be actively maintained and be capable of handling experimental replicates internally (which results in better specificity [8]). We also selected callers that have reasonable sensitivity and specificity characteristics when run in default settings as presented in published benchmarking studies, have reasonable operating characteristics when handling datasets with varying sequencing depths and signal-to-noise ratios, and have the ability to vary their peak scanning windows. Peak callers were also selected based on their input requirements, output file format, software dependencies, stability and computing resources required when running.

Our final criterion for candidate callers related to the implemented algorithm for peak calling. We selected peak callers that generate a statistical model of the background signal to ascertain a peak significance, as this has been shown to have superior performance to other approaches [35]. MACS2 [25] and HOMER [15], two commonly cited and well benchmarked peak callers despite their relative age [8], utilize Poisson distributions to generate background models prior to statistical testing for peak significance between ChIP and control samples. In contrast, GEM utilizes a binomial test and supplements this by also considering presence of enriched motifs in potential peaks [16]. As GEM is only suited for narrow transcription factor style peaks, for broad histone mark peaks, the alternate peak caller selected was SICER2, an updated release of the SICER peak caller [17]. SICER works by performing spatial clustering of enriched windows to identify broad peaks; an approach that has shown solid performance in recognizing broad peaks [17]. SICER is also a very well-cited and well performing broad peak caller despite its age. The final peak caller selected, Genrich, utilizes a unique approach as it assumes a background model with a log-normal distribution and calls peaks by testing whether the read pileups have a total area under the curve (AUC) above a significant threshold [13]. Owing to this disparate approach, we chose to include Genrich, as a relatively new peak caller despite lacking citations or formal benchmarking. In summary, we have primarily selected peak callers that show favorable performance in peer-reviewed, published benchmarking papers, as benchmarking of peak callers is beyond the scope of this publication.

### Analysis customization and reproducibility through the settings table

An on-going issue in science is irreproducibility of results [36]. In spite of journals enforcing stricter reporting of methods, ChIP-seq processing steps consistently lack essential details with omission of key program modification parameters or entire steps. To circumvent this and ensure ChIP-AP analysis reproducibility, we implemented a standardized ST. The ST lists all the programs used in ChIP-AP and their optional program arguments for an analysis. If no table is provided, ChIP-AP will use a default ST (DST; Figure 1**—**Input, Table 1), which we have compiled based on our testing and found to yield consistently adequate results. (Additional ST parameter tuning beyond the DST is advised only for advanced users who have consulted the documentation for the relevant programs.) To reproduce any analysis performed by ChIP-AP, users need only provide the ST and the raw FASTQ files. As such, the dissemination of the ST is essential alongside results. The ST can be included as a supplemental table or as a processed data file when submitting to public repositories. The ST ensures analysis reproducibility in a consistent and convenient format for published works.

**Table 1:** ChIP-AP default ST

Program	Argument
fastqc1	-q
clumpify	dedupe spany addcount qout=33 fixjunk
bbduk	ktrim=l hdist=2
trimmomatic	LEADING:20 SLIDINGWINDOW:4:20 TRAILING:20 MINLEN:20
fastqc2	-q
bwa_mem	
samtools_view	-q 20
plotfingerprint	
fastqc3	-q
macs2_callpeak	
gem	-Xmx10G --k_min 8 --k_max 12
sicer2	
homer_findPeaks	
genrich	--adjustp -v
homer_mergePeaks	
homer_annotatePeaks	
fold_change_calculator	--normfactor uniquely_mapped
homer_findMotifsGenome	-size given -mask
meme_chip	-meme-nmotifs 25

*Note*: The default ST used by ChIP-AP if no user provided table is provided. The left column lists the constituent programs of ChIP-AP with their optional modification parameters/flags found in the right column. A copy of the ST utilized by any ChIP-AP run can always be found in the analysis output folder.

### ChIP-AP graphical interfaces

Multi-step ChIP-seq analyses require coding experience and proficiency to be performed correctly. To cater to users with no coding experience, platforms such as Galaxy, or software suites such as Partek have been developed to make analyses accessible, albeit occasionally behind subscription-based services. However, for ChIP-seq analyses, these platforms utilize a single peak caller with limited customization options that can result in incomplete surveying of the binding landscape as previously noted. In contrast, ChIP-AP incorporates its own graphical interfaces to aid users in completing analyses and is open-sourced software.

To cater to the breadth of proficiencies in the scientific community, two interfaces have been implemented as part of ChIP-AP. The first interface, the Wizard (Figure 1 and Supplementary Figure 1A, available online at http://bib.oxfordjournals.org/), guides users through analysis configuration via a series of windows, each asking for a single input. For more experienced users, we implemented the Dashboard, which enables inputting all the required information from a single window (Figure 1 and Supplementary Figure 1B**—**Data Input, available online at http://bib.oxfordjournals.org/). Additionally, the dashboard contains a command line translation panel (Supplementary Figure 1B**—**Command Line Translation, available online at http://bib.oxfordjournals.org/) that dynamically updates to reflect additional/changed inputs enabling users to link graphical elements to command line arguments that control pipeline behavior. This implementation will aid some researchers more confidently transition from interface to command line usage of ChIP-AP. Independent of whether the wizard or dashboard is utilized, users will be questioned to use either a custom ST or the DST. This enables full customization of analyses independent of its mode of use.

## Results

### Consensus peaks increase the accuracy of motif and gene ontology analyses

**Figure 2:**
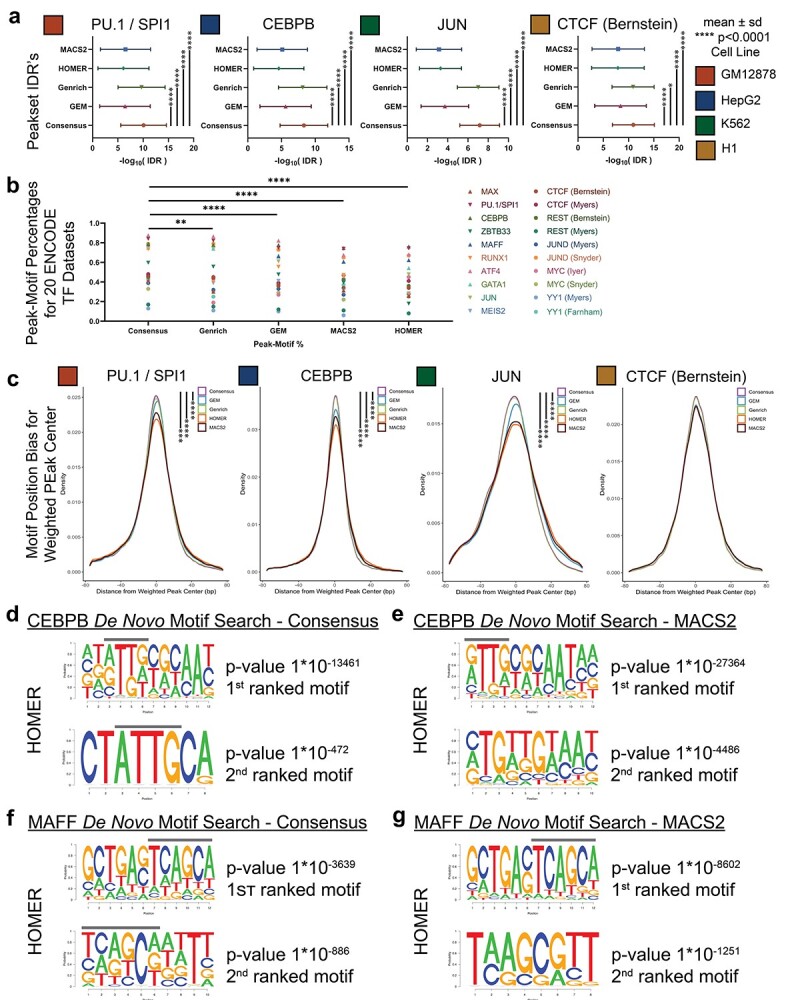
Consensus peak set improves detected motif accuracy. (**A**) IDRs for all peaks in each corresponding peak set. Shown in this panel is the mean IDR ± the SD. For statistical comparison, unpaired two-sided *t*-tests were performed on the distribution of −log_10_(IDR) values with ^*^^*^^*^^*^P < 0.0001. (**B**) Peak-motif percentage (the percentage of peaks with the canonical-binding motif) for all 20 TF’s profiled as identified for the consensus peak set and all individual peak callers. On average, the consensus peak set shows significant improvements in peak-motif percentages as compared to the individual callers [two tailed *t*-test’s; Consensus versus Genrich *P* = 0.009(^*^^*^), Consensus versus GEM *P* < 0.0001(^*^^*^^*^^*^), Consensus versus MACS2 *P* < 0.0001(^*^^*^^*^^*^), Consensus versus HOMER *P* < 0.0001(^*^^*^^*^^*^)]. (**C**) The motif position-biases for SPI1/PU.1, CEBPB, JUN and CTCF (Bernstein) for the consensus peak set and all individual callers. The position-bias is a measure of how far the identified motif sits away from the weighted peak center. *Z*-test statistics were performed on distributions of position distances with ^*^^*^^*^P < 0.001, ^*^^*^^*^^*^P < 0.0001; see Supplementary Table 2 available online at http://bib.oxfordjournals.org/. (**D**) The CEBPB *de novo* motif discovery results as reported by HOMER for the consensus peak set. The line above the peaks delineates position of the monomer binding motif. The second motif result clearly shows the canonical CEBPB monomer motif without homo- and hetero-dimer motif sequences. (**E**) The CEBPB *de novo* motif discovery results as reported by HOMER for the MACS2 peak set. The line above the peaks delineates position of the monomer binding motif. In contrast to the lower figure of panel (**D**), the monomer-binding sequence cannot be observed. (**F**) The MAFF *de novo* motif discovery results as reported by HOMER for the consensus peak set. The line above the peaks delineates position of the monomer binding motif. Both panels show the canonical MAFF monomer binding motif. (**G**) The MAFF *de novo* motif discovery results as reported by HOMER for the MACS2 peak set. The line above the peaks delineates the position of the monomer binding motif. In contrast to (**F**), the MACS2 results are not as consistent in showing the MAFF monomer binding motif.

A cornerstone of scientific research is result reproducibility through replication and/or independent techniques, which instill greater confidence in result validity. For this reason, peaks identified in multiple sample replicates are deemed more confident and have favorable IDR [27]. By extension, we propose that ChIP-seq peaks detectable by multiple peak callers, each utilizing independent detection algorithms, should therefore garner greater confidence and more favorable IDR scores, in addition to also being identified across (biological or technical) sample replicates. To assess whether the consensus peak set (the peaks concomitantly detected by all peak callers) identifies the most confident peaks, we analyzed 20 transcription factor (TF) datasets from differing TF families, across four cell lines from ENCODE [37] (Supplementary Table 1, available online at http://bib.oxfordjournals.org/). In ChIP-AP’s output table, alongside a detected peaks coordinates, we report a listing of the detecting callers, as well as the peaks IDR. When extracting the consensus peaks for each TF, we can validate that these peak sets indeed show the most significant IDR scores ensuring we are identifying the most confidently called peaks for each respective TF (Figure 2A and Supplementary Figure 2A, available online at http://bib.oxfordjournals.org/).

We next hypothesized that the consensus sets should also significantly improve peak-motif percentages (the percentage of peaks containing a valid binding motif) and, for certain datasets, the motif position bias (the distance of the motif to the weighted peak center), two measures of peak validity [38, 39]. To investigate the performance of the consensus peak set on peak-motif percentages, we compared the consensus peak set for each TF against the independent peak callers. For each of the selected TF’s, canonical-binding motifs were sourced from MethMotif [34] and the number of peaks with valid motifs was determined through a directed motif search using HOMER [15]. Across all TF’s, the consensus peak set showed a significant improvement in the average peak-motif percentage as compared to the independent peak callers (Figure 2B and Supplementary Table 1, available online at http://bib.oxfordjournals.org/).

Next, to assess whether the identified motifs were focused near the weighted peak center (not simply the peak center), the motif position bias for the consensus peak set was again, compared to the other independent peak caller sets. For some TF datasets, the consensus peak set provided the best motif position bias results such as in PU.1/SPI1, CEBPB, JUN and CTCF (Bernstein) (Figure 2C), while in other datasets we observed mixed results (Supplementary Figure 2B and Supplementary Table 2, available online at http://bib.oxfordjournals.org/). As this metric does not detract or alter peak-motif percentage rates, the performance of the consensus peak set in this regard while noteworthy, it is not essential or critical that the consensus peak set show the best performance in this metric. Surprisingly though, GEM was able to out-perform all other peak sets in certain datasets, likely due to its internal motif-centric approach to peak calling. Taken together though, our results show the consensus peak set can consistently and significantly improve peak-motif hit rates and, in some datasets, even improve on the motif position bias results. With such findings, we next hypothesized whether the consensus peak set can also increase the likelihood of identifying monomer and homodimer binding motifs in preference to heterodimer sequences which contain co-factor motif sequences.

Previously, Lin *et al.* [40] outlined how global motif analyses can contain heterodimer motif sequences, which include co-factor motif sequences, in addition to the target proteins monomer binding motif. In their research, they highlighted heterodimer motifs for CEBPB and MAFF when performing *de novo* motif analysis utilizing MACS2 called peaks. We therefore derived the ChIP-AP consensus peak sets for CEBPB and MAFF, and performed *de novo* motif searches using both HOMER [15] and the MEME-Suite [28, 41, 42] and compared findings of the consensus peak sets to the lone MACS2 peak set to unbiasedly determine the differences in motif detection capabilities. For CEBPB, the first motif candidate reported by HOMER for both peak sets were near identical, which, according to Lin *et al.* [40], contains a mixture of homo- and hetero-dimer sequences (Figure 2D and E, upper panels available online at http://bib.oxfordjournals.org/). However, the second motif for the consensus peak set (Figure 2D, lower panel available online at http://bib.oxfordjournals.org/) shows the CEBPB monomer ATTG binding motif, devoid of dimer sequences [40]. The second motif hit from the MACS2 peak set (Figure 2E, lower panel available online at http://bib.oxfordjournals.org/), fails to match this finding. When analyzing the consensus peak set using the MEME-Suite, the results showed two motifs with heterodimer sequences (Supplementary Figure 2C, available online at http://bib.oxfordjournals.org/, ranked 1st and 3rd), two motifs with the homodimer sequences (Supplementary Figure 2C, available online at http://bib.oxfordjournals.org/, ranked 2nd and 4th), and the fifth motif showed the monomer ATTG motif. Conversely, for the MACS2 dataset, when analyzed using the MEME-Suite, three motif results contained heterodimer sequences (Supplementary Figure 2D, available online at http://bib.oxfordjournals.org/, ranked 1st, 3rd and 4th), one result showed the homodimer sequence (Supplementary Figure 2D, available online at http://bib.oxfordjournals.org/, ranked 2nd) and the fifth result showed the monomer binding sequence. Therefore, for CEBPB, both algorithms reported similar findings, yet the consensus peak set showed a more direct signal for the CEBPB monomer binding motif, a finding not immediately evident from the MACS2 peak set without careful inspection.

Similar results to CEBPB were also found for the TF, MAFF. For the MAFF consensus peak set, the top two HOMER *de novo* motif results were consistent and showed the TCAGCA binding sequence (Figure 2F). The MACS2 peak set however, showed differing sequences for the top two hits (Figure 2G). When analyzed using the MEME-Suite (Supplementary Figure 2E and F, available online at http://bib.oxfordjournals.org/), MEME consistently calls the TGCTGA motifs for both peak sets, but the sixth reported motif candidate for the consensus peak set shows a homodimer binding profile, a result not recapitulated in the MACS2 peak set entirely. The consensus peak set is therefore capable of guiding *de novo* motif discovery analyses to identify monomer and homodimer binding profiles without needing to compare and overlap transcription factor and co-factor datasets in a manner as described by Lin *et al.* [40].

As the consensus peak set had thus far consistently shown improved performance when assessing motif presence, we questioned whether the consensus set can also aid in improving GO investigations by ensuring only the strongest binding candidates are included in analysis that should therefore result in higher ranking of more biologically related terms. To test the efficacy of the consensus peak set as compared to the other called peak sets, we compared GO analyses for all assessed TFs. We observed that for the RUNX1, ATF4, JUN, ZBTB33 and GATA1 datasets, the GO results for the consensus peak sets returned more functionally relevant and related terms than the MACS2 called peak sets (Supplementary Tables 3 and 4, available online at http://bib.oxfordjournals.org/). In particular for RUNX1, the top 20 MACS2 GO results contained generic terms while the consensus peak results clearly outlined RUNX1 functions in hematopoietic differentiation, regulation of metabolic and signaling pathways, and autophagy regulation, all of which are known functions of RUNX1 [43–48] (Supplementary Table 5, available online at http://bib.oxfordjournals.org/). Working from this observation, we looked at the top 50 GO terms called by the consensus peak set and plotted to see how comparably ranked each GO term was across all other individual peak sets. This revealed that most GO terms were ranked quite differently in the consensus peak set as compared to other peak sets (Supplementary Figure 2G, Supplementary Table 6, available online at http://bib.oxfordjournals.org/). Careful manual inspection of the disparate rankings showed that in datasets such as for RUNX1, ATF4 and JUN, a noticeably higher ranking of more targeted terms is evident as compared to the individual callers. Unfortunately, this observation was not universal and the rankings observed in other datasets (such as for CTCF and REST (Myers)) was comparable to those of any individual caller. Therefore, in best case scenarios, the consensus peak GO terms can provide higher ranking of targeted and more direct terms owing to its focus on the only the most confident gene targets, while in less well-performing datasets, the reported findings are comparable to those of any individual peak caller giving rise to a ‘no-lose’ scenario for users that can be of significant benefit if investigating less well-characterized proteins.

To conclude then, we have shown that utilizing the consensus peak set is able to provide significant benefits and improvements in peak-motif percentages, motif position bias, improving *de novo* motif discovery, and more directly identify biologically significant ontology terms. Overall, the consensus peak set can provide significant improvement over using a single peak caller when the biological question posed is to investigate the most confident targets of DNA-binding protein.

### ChIP-AP peak set robustness

**Figure 3:**
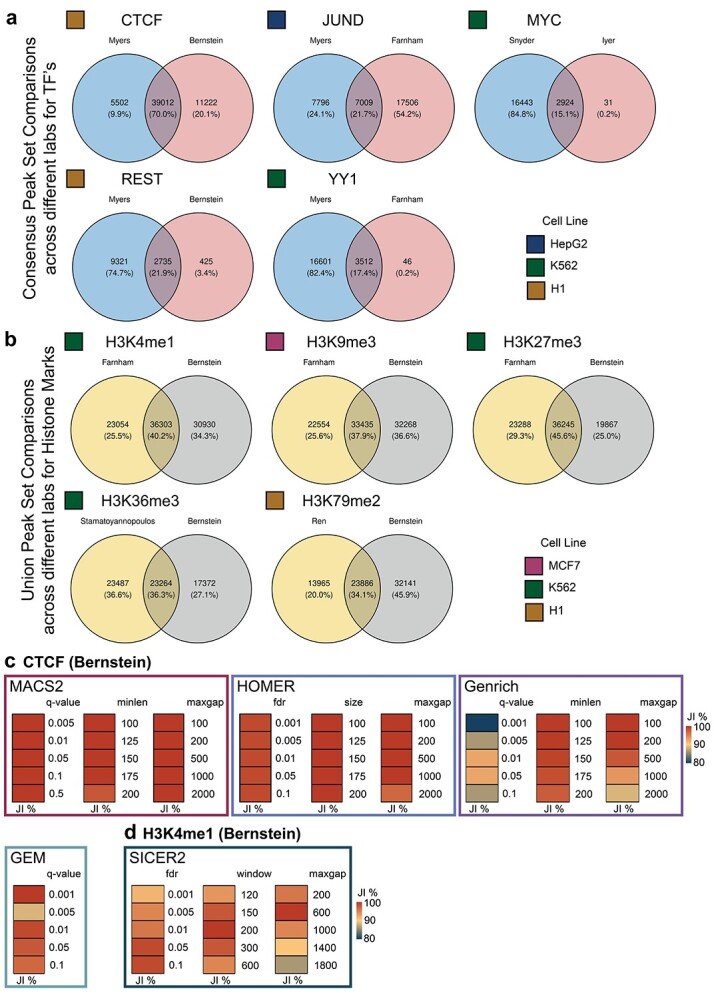
ChIP-AP peak set robustness. (**A**) Venn diagrams highlighting the number and percentage of consensus peak set overlapping peaks of inter-lab ChIP-seq datasets. Two datasets were obtained from ChIP-seq experiments on the same DNA-binding proteins of interest in the same cell lines but performed and uploaded to the ENCODE project database by different labs. Five pairs among the most common ChIP-seq transcription factor datasets were picked for this analysis. All datasets were processed with ChIP-AP in default settings and their resulting consensus peak sets were merged in a pair-wise manner. This panel shows venn diagram depicting the consensus peak set overlap between each pair of the five transcription factor dataset pairs (CTCF, JUND, MYC, REST and YY1). (**B**) Venn diagrams highlighting the number and percentage of union peak set overlapping peaks of inter-lab ChIP-seq datasets. Two datasets were obtained from ChIP-seq experiments on the same histone marks of interest in the same cell lines but performed and uploaded to the ENCODE project database by different labs. Five commonly studied histone mark datasets were picked for this analysis. All datasets were processed with ChIP-AP in default settings and their resulting consensus peak sets were merged in a pair-wise manner. This panel shows venn diagrams depicting the union peak set overlap between each pair of the five histone marker dataset pairs (H3K4me1, H3K9me3, H3K27me3, H3K36me3 and H3K79me2). Above of each set is the name of the lab from which the dataset was derived. (**C**) Heatmaps depicting the consensus peak set Jaccard Index score when one peak calling parameter was modified, compared to when the peak calling parameters are all at default values. Comparable parameters across different peak callers were modified and compared. Heatmap color range for the Jaccard Index extends between 80 and 100% overlap to better show variability in differences. High JI’s represent a high degree of overlap between the modified parameter peak set and the default consensus peak set.

In order to increase confidence in identified peaks, typically sample replicates are performed and only consistently called peaks are selected. ChIP-AP’s consensus peak set takes this approach further and collates peaks called by independent peak callers to add an additional metric assessing peak confidence, beyond replicates. To validate the robustness of this approach, in addition to the previously discussed and commonly accepted peak quality metrics, we analyzed five well-studied TF’s (CTCF, JUND, MYC, REST and YY1), and compared the consensus peak sets across two datasets performed by independent labs in the same cell line (see Methods) to see how consistent the consensus peak set can be; all the while knowing that the wet-lab variability across two independent labs will be a significant source of variability and difference between the datasets. Reassuringly, in spite of the significant inter-lab variability observed (the significantly different number of called peaks between the labs), the overlap of the consensus peak sets was quite robust with consistent observations of the consensus peak set from one lab being near entirely identifiable in the second lab’s dataset, particularly for MYC, REST and YY1 (Figure 3A). It is evident from this comparison however the inter-lab variability can significantly affect peak set consistency such as the case with CTCF and JUND. To somewhat mitigate this variability, one can instead opt to focus on the union peak set (all peaks called by all callers) and utilize that for comparison that can potentially yield an increased number of overlapping peaks (Supplementary Figure 3A, available online at http://bib.oxfordjournals.org/). However, where the union peak set overlap for inter-lab datasets would be more suited, would be when comparing histone marks. Whereas TF often have defined binding sites that contain a canonical binding motif, histone marks may have very broad peaks, or even a mixture of peak types [12] that can change depending on a cells state and culture conditions. In such datasets, where the histone mark deposition sites can change depending on numerous variables, selecting all identifiable enrichment sites seems conceptually more encompassing. By comparing histone mark enrichment sites utilizing the union peak sets, we can still see good overlap across inter-lab experiments (Figure 3B).

With ChIP-AP incorporating multiple peak-callers, one can question how altering each caller’s behavior (by modifying command line parameters) can alter peak set overlaps, particularly the consensus peak set. To investigate this, for the Bernstein lab derived CTCF dataset, we generated the consensus peak set using the ST provided (in Methods), and then we proceeded to test how modifying command line parameters altered the default consensus peak set by calculating the Jaccard Index (JI) between the modified and the default consensus peak sets. Where possible, we aimed to modify comparable parameters between peak callers and tested five reasonable values for each parameter. For MACS2, we modified the parameters controlling maximum peak *q*-value (-q), minimum peak length (--min-length) and maximum distance between two peaks (--max-gap). For HOMER, we modified the parameters controlling maximum peak False Discovery Rate (-fdr), peak size (-size) and maximum distance between two peaks (-minDist). For Genrich, we modified the parameters controlling maximum peak *q*-value (-q), minimum peak length (-l) and maximum distance between two peaks (-g). For GEM, we modified the parameter controlling maximum peak *q*-value (--q), which is the only one parameter in GEM that is comparable to the tested parameters in the other three peak callers. Finally, to assess how changing parameters alters peak calling for a histone mark set, we tested altering parameters for SICER2 on the H3K4me1 Bernstein lab derived dataset (see Methods). For SICER2, we modified the parameters controlling maximum peak False Discovery Rate (-fdr), peak scanning window size (-w) and maximum distance between two peaks (-g).

Looking first at the CTCF dataset, we can see that modifying MACS2 parameters (Figure 3C**—**left panel), resulted in negligible changes in JI values, with each modified consensus peak set showing near perfect overlap with the default consensus peak set irrespective of the parameter values. Likewise, we also see a similar trend for HOMER’s parameters (Figure 3C**—**middle panel) where modifications negligibly altered the consensus peak set result. On the other hand, we can see noticeable changes in the consensus peak sets when altering Genrich’s *q*-value parameter (Figure 3C**—**right panel). Modifying Genrich’s minimum peak length and maximum distance between two peaks show mildly greater variability compared to MACS2 and HOMER, but not to the same extent as modifying the *q*-value parameter (Figure 3C**—**right panel). Finally, similar to what we see when modifying MACS2 and HOMER parameters, the resulting consensus peak set is rather stable irrespective of the set parameter values for GEM (Figure 3C). However, we noted a quirk of GEM during testing. When run in multi-threaded mode, no two runs yielded identical outputs even if run with the same parameters, there will always be minor differences, a point that has been raised on the GitHub repository. To avoid this, GEM has to be run in single-threaded mode to ensure reproducible results across runs.

Since the peak callers tested for a TF dataset all showed consistent results, for the H3K4me1 dataset we only assessed the performance of SICER2 (Figure 3D). In these results, we can see that in contrast to the other peak callers, altering the command line parameters of SICER does yield mildly greater variability in the resulting consensus peak set, but one must also note that the JI’s reported are all still approximately 90% or greater. Overall, we have observed that among all the parameters we tested across all peak callers incorporated into ChIP-AP, modification of individual settings does not drastically alter the resulting consensus peak set, highlighting the consistency and robustness of ChIP-AP’s consensus peak despite possible parameter variabilities in each individual peak caller.

### Capturing lost peaks with the union peak set

Every peak caller has differing operating characteristics and abilities to handle poorly enriched or high signal-to-noise datasets [8, 12]. Single peak caller analyses are solely dependent on the ability of that chosen caller to handle that dataset’s enrichment profile, which if handled unsuccessfully, will result in few peaks being called giving an inconclusive result. However, other peak callers can be more capable at handling poorly enriched datasets. Therefore, the dataset needs to be analyzed with the right peak caller for its specific characteristics, the choice of which may not be evident in advance.

**Figure 4:**
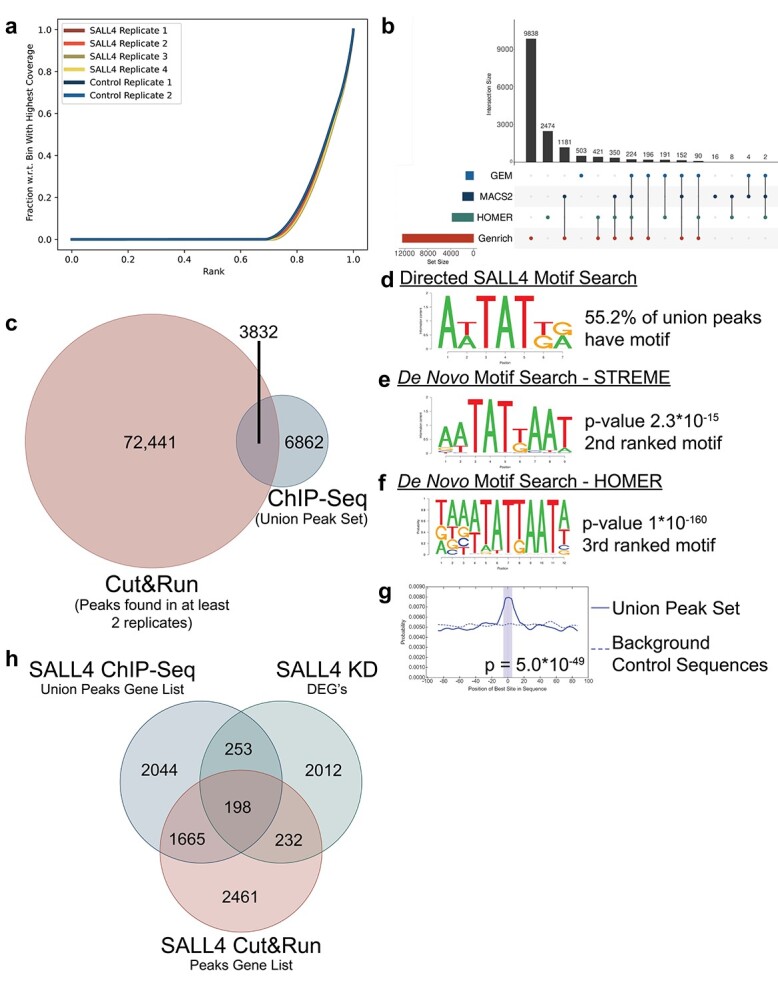
Union peak set comprehensiveness and accuracy. (**A**) Fingerprint plot for aligned sequence files. Negligible separation between the SALL4 and control curves indicates poor enrichment in the SALL4 samples, an experiment typically considered as having failed. (**B**) Upset plot describing the distribution of peaks observed by each peak caller. The left histogram represents the total number of called peaks per caller. The top histograms represent the size of the sub-sets in question. The connected circles represent highlighted overlap. (**C**) Venn diagram showing the overlapping number of peaks between the SALL4 union ChIP-seq dataset and the Cut&Run dataset, both of which were done on SNU-398 cells. (**D**) The motif sequence used for the directed motif search in the SALL4 ChIP-seq union set, which was found in 55.2% of the union set. (**E**) The STREME *de novo* motif search for the SALL4 union peak set identified the AT-rich binding motif as the second result. (**F**) The HOMER *de novo* motif search for the SALL4 union peak set identified the AT-rich binding motif as the third result. (**G**) The STREME identified motif (shown in **D**) was found centrally enriched in the union peak set as compared to background sequences; an accepted indicator of a valid motif result. (**H**) Venn diagram depicting the degree of overlap in the number of genes between the SALL4 ChIP-seq union peak set, the SALL4 Cut&Run peak set and the SALL4 KD DEG list.

The oncogene sal-like protein 4 (SALL4) maintains pluripotency and the self-renewal characteristics of embryonic stem cells [49], is typically down-regulated following birth, and is aberrantly expressed in many tumors [49, 50]. SALL4 has multiple protein interacting partners along with DNA-binding and regulation functions [31, 49]. SALL4 has also been difficult to profile using ChIP-seq. A SALL4 ChIP-seq was performed on SNU-398 cells with results showing poor enrichment evidenced by little separation between the SALL4 replicates and control enrichment curves (Figure 4A). When processed with ChIP-AP, GEM and MACS2 struggled to call peaks, returning 1362 and 1937 peaks, respectively (Figure 4B). HOMER called approximately double the number of peaks (3760), but Genrich was able to call 12 452 peaks. Notable also was the sporadic overlap between called peak sets (Figure 4B).

To extract as much data as possible from the performed ChIP-seq, we investigated whether the union peak set (all called peaks) (Supplementary Table 7, available online at http://bib.oxfordjournals.org/) contained valid SALL4 targets by comparing to a SNU-398 SALL4 Cut&Run dataset [31]. Cut&Run utilizes antibody-targeting and micrococcal nuclease digestion to map DNA-binding sites [51]. It is an analogous yet independent technique to ChIP-seq, thereby providing a comparison dataset. For the Cut&Run peak set, we selected peaks found in at least two replicates (three biological replicates total) and that showed at least a 4-fold enrichment over IgG. When comparing the ChIP-seq union peak set to the Cut&Run peaks, we observed a 36% peak overlap in the union peak set, a comparable percentage to having used an individual peak caller (Supplementary Figure 4A, available online at http://bib.oxfordjournals.org/) but with more peaks (Figure 4C), thus facilitating a more comprehensive survey of the binding landscape by circumventing individual poor performance.

In addition to the Cut&Run comparison, we interrogated the union peaks for the presence of the SALL4 DNA-binding motif [31]. A directed motif search was performed on the union peak set that revealed 55% of peaks contained the SALL4 motif (Figure 4D). To unbiasedly investigate motif presence, *de novo* motif searches were undertaken using both the MEME-Suite [28, 41, 42] (which incorporates STREME, CentriMo and MEME-ChIP) and HOMER, which called the SALL4 binding motif as the second and third candidate hits respectively (Figure 4E and F) with MEME-ChIP calling the motif as the second and third candidates as well (Supplementary Figure 4B, available online at http://bib.oxfordjournals.org/). Additionally, CentriMo reported the STREME identified motif was centrally enriched (Figure 4G), an expected observation for true binding motifs [38]. Therefore, despite utilization of all called peaks, the union peak set still showed enrichment of the SALL4 binding motif.

To validate the inclusion of valid SALL4 target loci in the union peak set, the peak corresponding gene list was compared to a SALL4 knock-down differentially expressed genes list (SALL4 KD DEG) [31]. It was reported that SALL4 KD resulted in differential expression of 2695 genes, 430 of which had a corresponding Cut&Run peak (Figure 4H). Comparing the SALL4 KD to the union peak set showed an overlap of 451 genes, 198 of which were shared with the Cut&Run overlapping genes (Figure 4H and Supplementary Table 8, available online at http://bib.oxfordjournals.org/). Additionally, SALL4 KD was found to increase the number of up-regulated genes in the ‘transcriptional regulator activity’ (GO:0140110) pathway, establishing pathway members as *bona fide* SALL4 targets [31]. Consistently, the union peak set GO analysis identified the same pathway as a top 20 enriched pathway (Supplementary Table 9, available online at http://bib.oxfordjournals.org/), with more significantly enriched GO terms pointing to SALL4 being a DNA-binding protein, a well-documented function [49, 50] (Supplementary Table 9, available online at http://bib.oxfordjournals.org/).

Overall, despite the poor enrichment observed in the SALL4 ChIP-seq, the union peak set contained valid data that could be extracted, used and validated by independent approaches [31]. The union peak set trades a marginal sacrifice in specificity for a significant gain in sensitivity, enabling the confirmation and presence of peaks identified or validated using different methodologies in sub-optimally enriched datasets. Whereas single peak caller analyses would produce inconclusive results, reliance on multiple peak callers can provide a significant improvement in reported findings and salvage sub-optimal datasets.

## Discussion

Over 50 peak callers have been published for analyzing ChIP-seq datasets each having distinct selectivity and specificity characteristics that are often not additive and seldom completely overlap in many scenarios even after modifying peak caller parameters. A peak caller can show superior performance in certain datasets, but not in others. Therefore, owing to the heterogeneity observed from profiling different DNA-binding proteins with differing immunoprecipitation and library preparation protocols, reliance on a single peak caller is unlikely to yield the most reliable, consistent, or comprehensive report of a proteins binding profile. To circumvent this, we developed ChIP-AP to integrate the results of multiple peak callers, thereby improving peak calling confidence and comprehensiveness to survey the binding landscape of DNA-binding proteins without requiring additional wet-lab observations. In our approach, the different peak callers utilized allow us to gauge peak reproducibility in a manner akin to utilizing replicates for *in vitro* findings or matched sets.

Many publications utilize receiver operating characteristic (ROC) curves when comparing peak caller results. Essential for this approach is having a defined ‘ground-truth’ or ‘gold-standard’ to compare back to. However, in light of numerous benchmarking studies that show significant differences between peak callers, no two peak callers interrogate the same peak calling space owing to different algorithmic selectivity and specificity characteristics. Furthermore, there is no gold-standard dataset that has had every peak validated using biochemically or *in vitro* means. We therefore argue that comparative approaches such as ROC may not give accurate results since there is no defined ground-truth to compare back to, and, that each peak caller reports a different set of peaks. As such, there is no equivalent findings to compare between called peak sets using this approach and it is for this reason that the results presented herein do not contain such comparisons. As presented though, we have opted to instead utilize IDR’s. By utilizing the IDR framework, we assess the reproducibility of each peak in the full peak list (union peak set) based on its detectability by each individual peak caller, and then sum the –logIDR values to generate a peak reproducibility rate. In this manner, concomitantly called peaks show greatest reproducibility, which is an expected result, since a peak has a greater likelihood of being a true-positive if it can be called by multiple, independent peak callers. In our testing of numerous datasets, we saw that peak reproducibility by independent peak callers to be a more telling of a peak validity than an attached statistic or numerical value. Despite the differences between peak callers however, they all report valid results that can describe real biology and no peak caller is universally superior over others. This is why ChIP-AP is implemented in a way so as to allow easy sub-setting of the called bound landscape, without needing reanalysis of data, to best address the biological question posed by the investigator. By utilizing the consensus peak set, binding motif accuracy can be significantly increased by restricting the motif search space to only the most confident peaks that are called concomitantly by multiple peak callers and have the most favorable IDRs. This can result in significantly improved peak-motif percentages in many circumstances as we observed. This can also improve outcomes of downstream GO analysis wherein more biologically significant terms can be reported. Furthermore, by utilizing the consensus peak set, motif enrichment results more readily report monomer and homodimer binding sequences as compared to heterodimer motif sequences. While all binding profiles are valid biologically, when performing a ChIP-seq on a specific transcription factor, like CEBPB, one would argue that the optimal motif result is the monomer and homodimer sequences of CEBPB alone, rather than observing heterodimer binding sequences that also contain co-factor sequences. This result should also be readily identifiable without necessitating overlapping multiple cell-line and co-factor datasets in order to subtract out co-factor motif sequences. The consensus peak set facilitates this from a single experimental analysis.

We next looked at the robustness of the consensus peak set by comparing results of two labs looking at the same TFs in the same cell lines. Our findings showed that while inter-lab variability had drastic effects on the peaks called, there were peaks that could be recapitulated between the two compared consensus peak sets, with the smaller dataset residing near entirely in the larger dataset. While in our investigations we looked at two datasets from different labs, such comparisons might be more suited, for example, when comparing different antibody batches within a single lab to compare the experimental findings of the same investigator, or when comparing two modifications of the same experimental protocol. In such scenarios, comparisons of either the consensus or the union peak sets could be quite insightful on the differences between sequencing runs and would be more insightful and biologically relevant than the results presented herein.

In stark contrast to the consensus peak set of a good quality dataset, if the profiled dataset has unfavorable sequencing characteristics such as poor enrichment, the union peak set can potentially yield improved results and allow users to marginally sacrifice specificity for a potentially significant gain in sensitivity. Utilizing the union peak set can potentially salvage an experiment and still identify biologically valid findings, despite the sub-optimal experimental efficiency. In between the two extremes of the consensus and union peak sets, is a gradient of thresholds selectable depending on the biological question or the presence of additional supportive data. The breadth of this gradient though is very much sample dependent. As we showed in selected datasets, changing peak caller parameters yielded negligible to minor differences in the final resultant consensus peak set. However, there will be cases where such parameter modifications yield drastic changes to the final result. Throughout our testing though, we noticed that the default parameter values of the selected peak callers are quite robust and are able to handle differing sample characteristics with little to no modifications. This allows users of ChIP-AP to focus on the biology by interpreting the presented, comprehensive binding landscape without needing to iteratively modify program parameters through repeat cycles of data reanalysis. While the results presented herein are for more well characterized datasets with clearer findings, one can foresee significant gains to be had in datasets for less well characterized proteins and factors where less literature evidence exists. In those circumstances, drawing on an analytical approach that yields more refined results would be of great benefit to investigators. ChIP-AP can therefore provide both substantial improvements to peak capturing and reliability from a single integrated and comprehensive analysis.

In conclusion, ChIP-AP is a seamlessly integrated end-to-end ChIP-seq analysis pipeline that is simple to use yet tremendously customizable through the ST. ChIP-AP is the first integrated end-to-end solution that utilizes multiple peak callers for ChIP-seq analyses that is accessible to both biologists, through the graphical interfaces, and bioinformaticians, through command-line usage. Our findings support the notion that ChIP-AP can help investigators better interrogate their experimental findings delivering different types of analyses of great value to those interested in transcriptional regulation in a manner that current single-caller approaches cannot provide in an accessible and easy to use manner.

**Key Points**

- ChIP-AP is the first integrated ChIP-seq pipeline that performs all analysis steps (raw FASTQ to final result) and seamlessly integrates four peak callers.
- By utilizing multiple peak callers, ChIP-AP allows users to analyse their data once, then apply filtering thresholds to answer different experimental questions, such as what are the best consensus binding sites? Or what are all the possible gene targets?
- ChIP-AP is targeted and tailored for biologists as it incorporates easy to use graphical interfaces while still retaining command-line usage for power users such as bioinformaticians.
- If selected, the union peak set (all called peaks by all callers) can salvage lost/missed peaks in low enrichment and high signal:noise datasets.
- If selected, the consensus peak set (concomitantly called peaks) significantly improves called peak confidence and improves peak-motif percentages.

## Supplementary Material

Supp_figure1_chipap_Large_bbab537

Supp_figure2_cosnensus_peaks_(Large)_bbab537

Supp_figure2_cosnensus_peaks2_(Large)_bbab537

Supp_figure2_cosnensus_peaks3_(Large)_bbab537

Supp_figure2_cosnensus_peaks4_(Large)_bbab537

Supp_figure3_result_robustness_Large_bbab537

Supp_figure4_capturing_lost_peaks_Large_bbab537

SuppTable1_bbab537

SuppTable2_bbab537

SuppTable3_bbab537

SuppTable4_bbab537

SuppTable5_bbab537

SuppTable6_bbab537

SuppTable7_bbab537

SuppTable8_bbab537

SuppTable9_bbab537

Supp_figure2_cosnensus_peaks5_(Large)_bbab537

## Data Availability

The sequencing and processed files for the SALL4 ChIP-seq have been uploaded to GEO with accession number GSE172355 (reviewer access token ilynoasgzperlgf). The ChIP-seq data are available to view using UCSC by adding the hub—http://137.132.97.62/public_hubs/mbassal/sall4_chipseq_ucsc_hub/hub.txt. Peaks referenced in this manuscript were called ‘sall4_merged_replicates’ over ‘control_merged_replicates.’ All other data utilized are publicly available with associated accession numbers provided.

## Code availability

ChIP-AP source code is available through the GitHub repository (https://github.com/JSuryatenggara/ChIP-AP). All additional commands utilized are outlined in the methods.

## Notes

**Jeremiah Suryatenggara** is a PhD student at the Cancer Science Institute of Singapore, National University of Singapore, with background in biomedical engineering. His current research focuses on developing integrated analysis tools and methods for NGS data of DNA/RNA-binding experiments.

**Kol Jia Yong** is a Senior Research Scientist at the Cancer Science Institute of Singapore and A^*^STAR with a background in gene regulation and cancer research. Her research interest is to study and develop early intervention for cancer and inflammatory diseases.

**Danielle E. Tenen** is a Senior Manager, Laboratory Operations at the Broad Institute of MIT and Harvard, with a background in technology development of genome-wide sequencing and analysis tools. She currently leads operation support for the twelve research teams within the Broad Institute's Cancer Program.

**Daniel G. Tenen** is a Principal Investigator at the Cancer Science Institute of Singapore, and a member of the Harvard Stem Cell Institute, Harvard Medical School. His major interest over four decades is gene regulation in normal cells and cancer.

**Mahmoud A. Bassal** is Post-Doctoral Research Fellow trained in both the biological and computer sciences at the Harvard Stem Cell Institute and the Cancer Science Institute of Singapore. His research interests focus on altered metabolism and epigenetics in cancer and disease, as well as developing novel analysis tools and integrative methods for data analysis.
