# Supplementary figures and images for: ChIP-AP: an integrated analysis pipeline for unbiased ChIP-seq analysis
Source: Brief Bioinform. 2021 Dec 30;23(1):bbab537. doi: 10.1093/bib/bbab537 (PMC8769893; doi:10.1093/bib/bbab537)

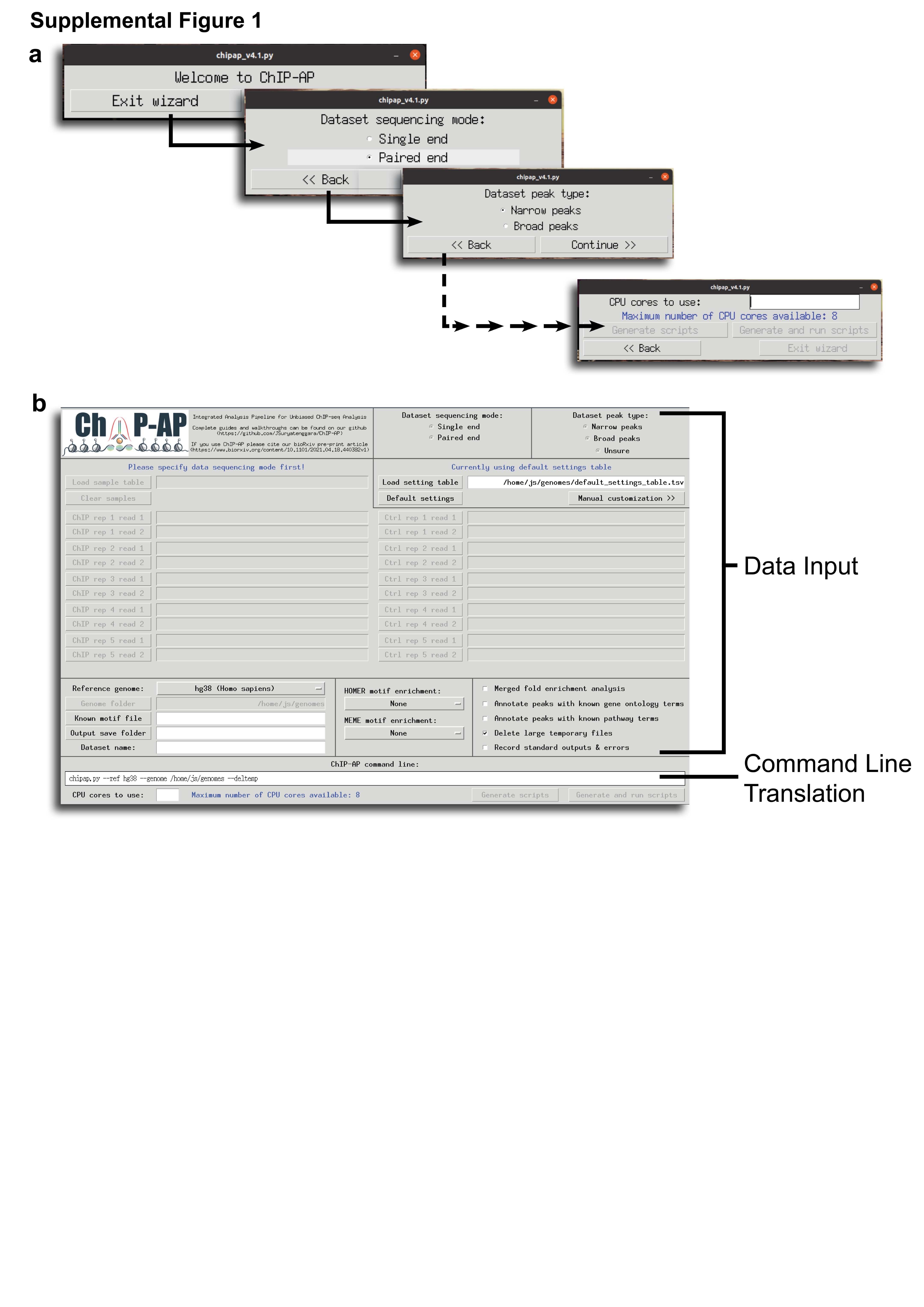

Supplement: Supp_figure1_chipap_Large_bbab537 [file supp_figure1_chipap_large_bbab537.jpeg]

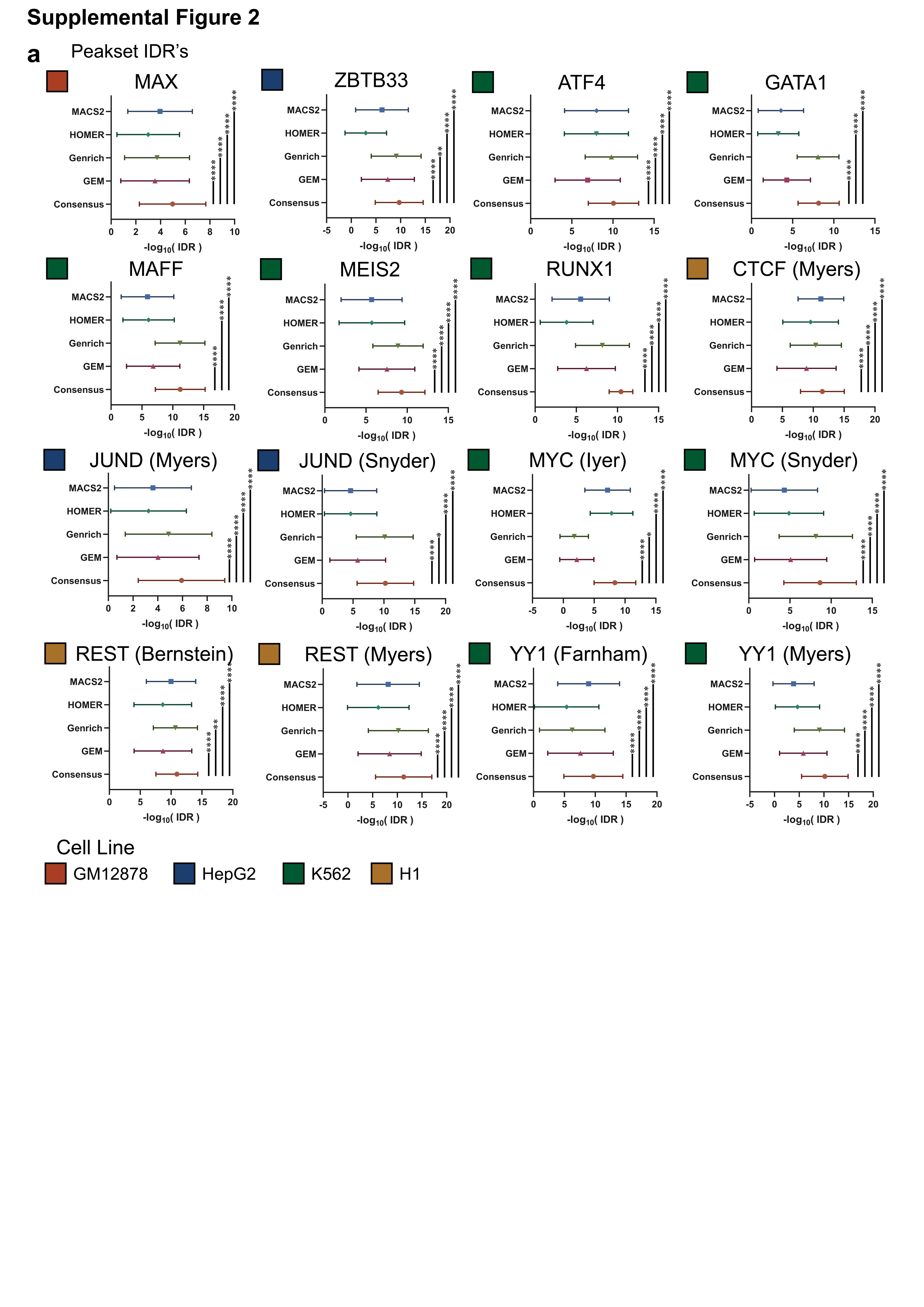

Supplement: Supp_figure2_cosnensus_peaks_(Large)_bbab537 [file supp_figure2_cosnensus_peaks_(large)_bbab537.zip › Supp_figure2_cosnensus_peaks_(Large)_bbab537.jpg]

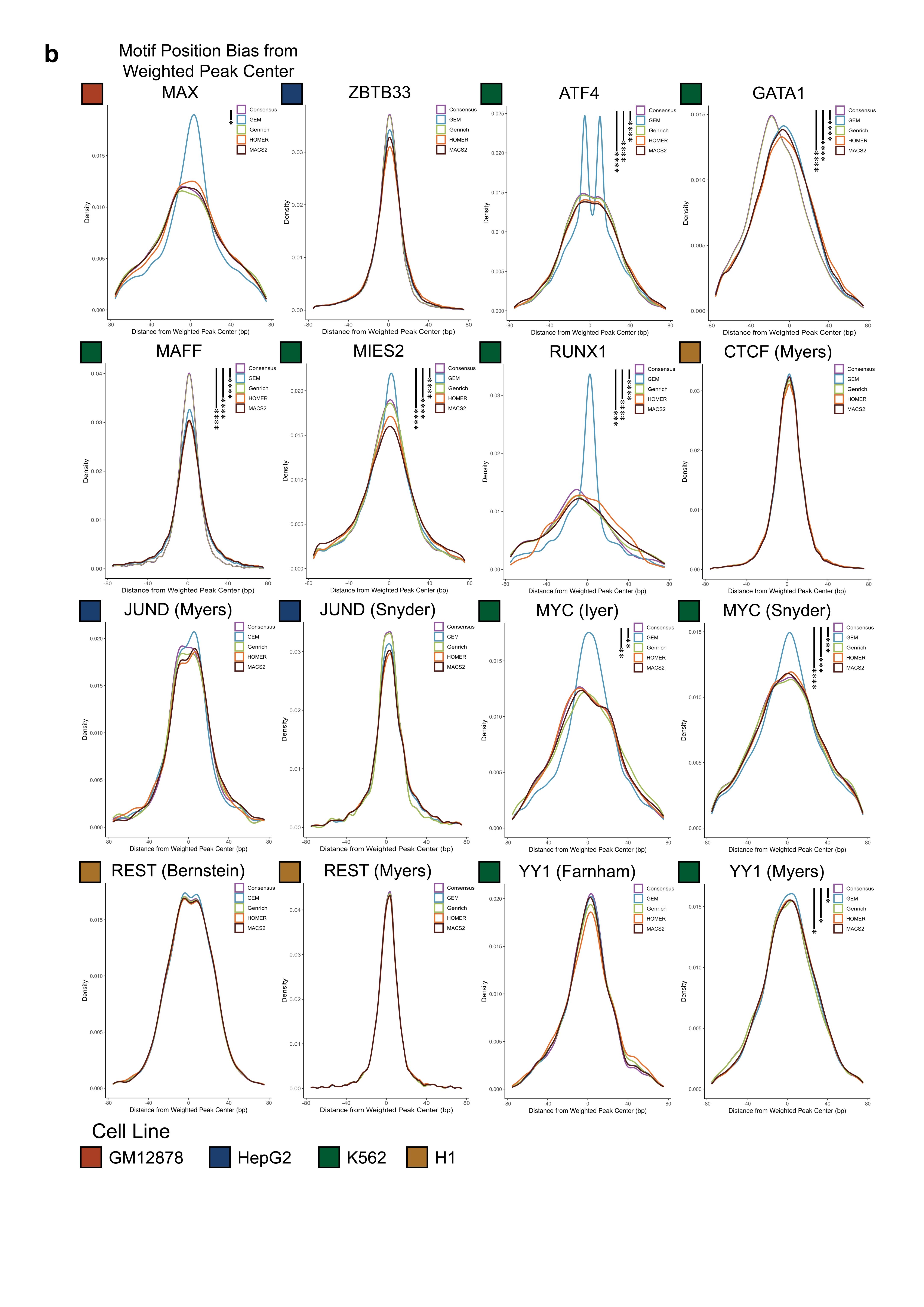

Supplement: Supp_figure2_cosnensus_peaks2_(Large)_bbab537 [file supp_figure2_cosnensus_peaks2_(large)_bbab537.zip › Supp_figure2_cosnensus_peaks2_(Large)_bbab537.jpg]

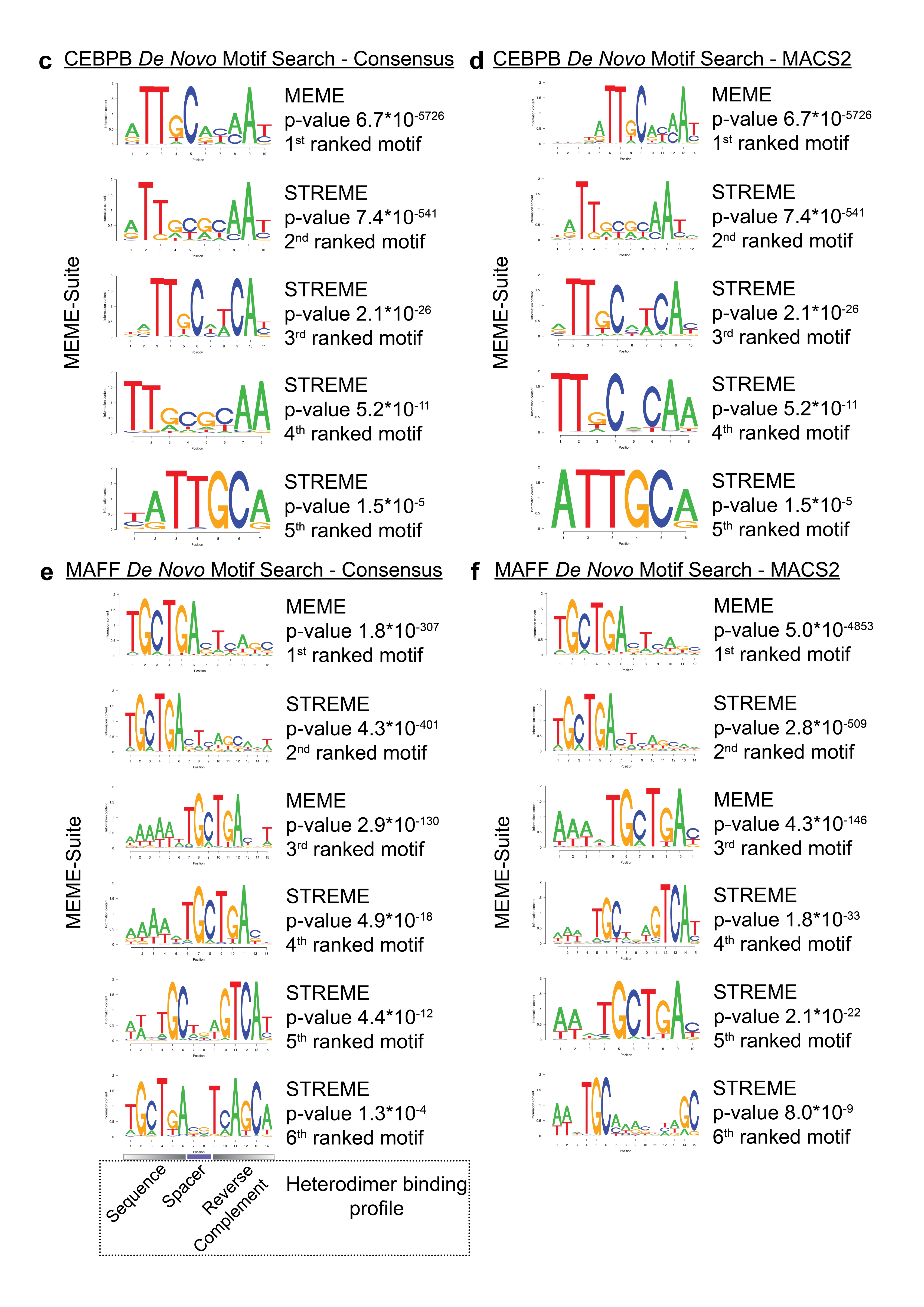

Supplement: Supp_figure2_cosnensus_peaks3_(Large)_bbab537 [file supp_figure2_cosnensus_peaks3_(large)_bbab537.zip › Supp_figure2_cosnensus_peaks3_(Large)_bbab537.jpg]

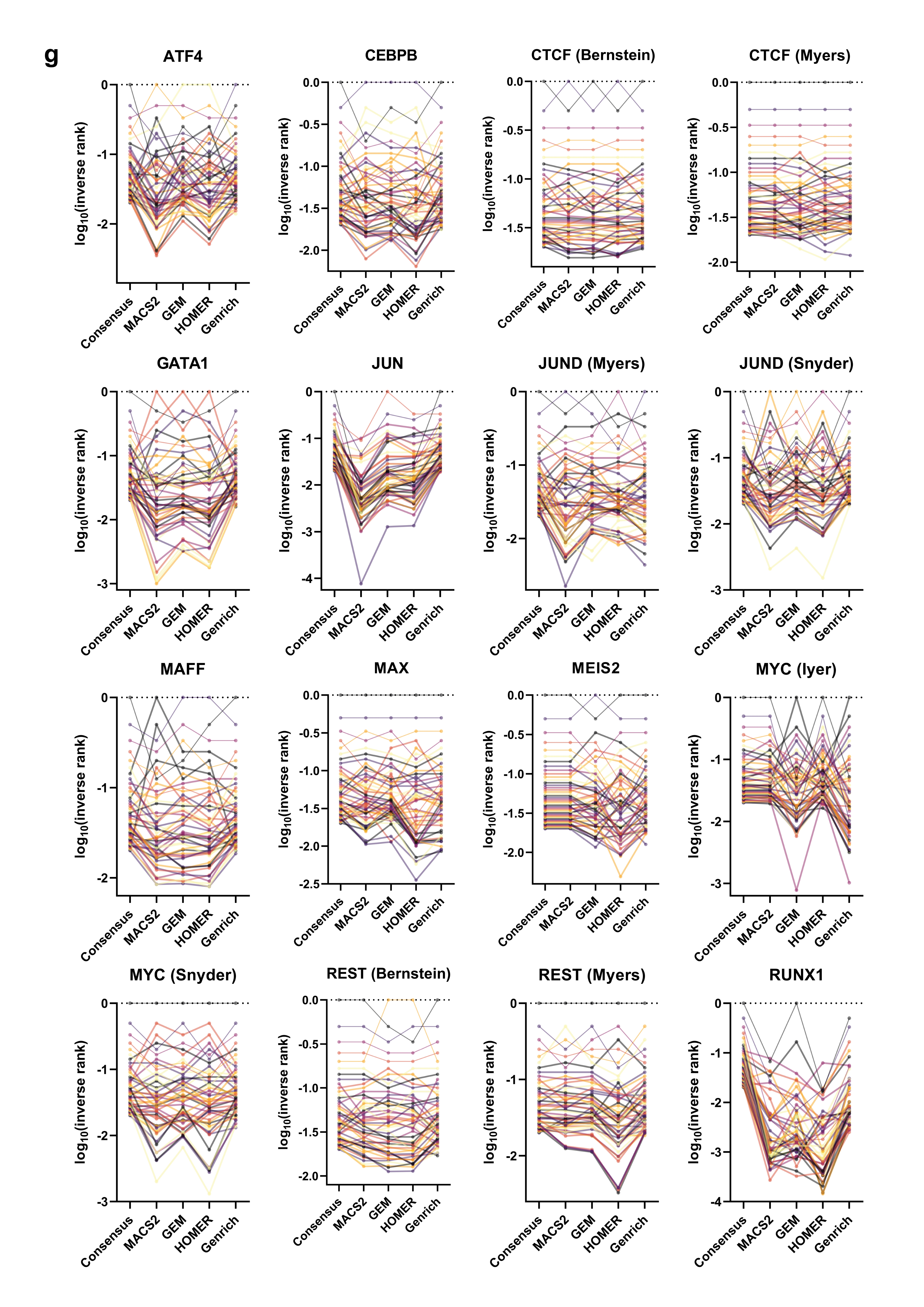

Supplement: Supp_figure2_cosnensus_peaks4_(Large)_bbab537 [file supp_figure2_cosnensus_peaks4_(large)_bbab537.zip › Supp_figure2_cosnensus_peaks4_(Large)_bbab537.jpg]

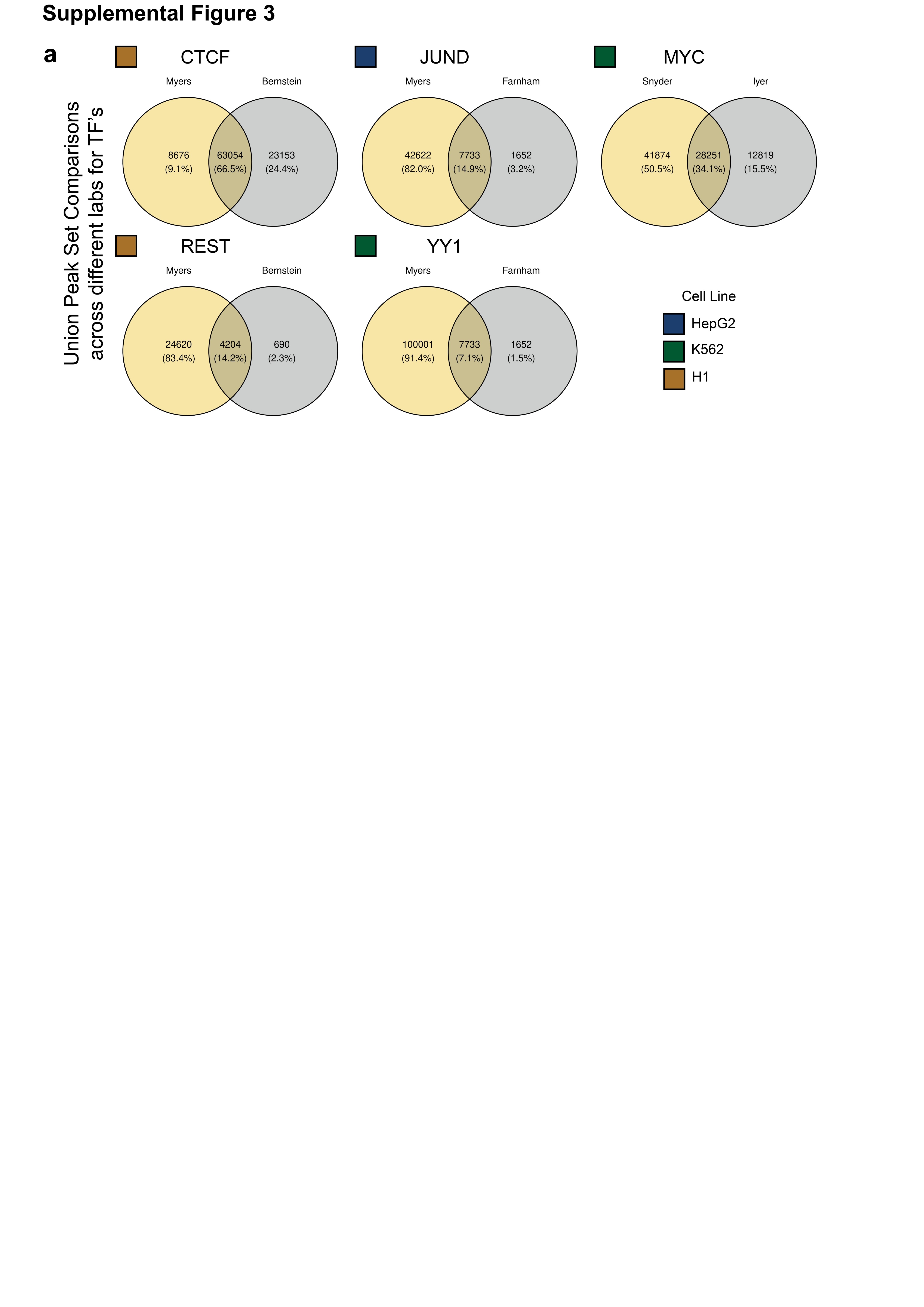

Supplement: Supp_figure3_result_robustness_Large_bbab537 [file supp_figure3_result_robustness_large_bbab537.jpeg]

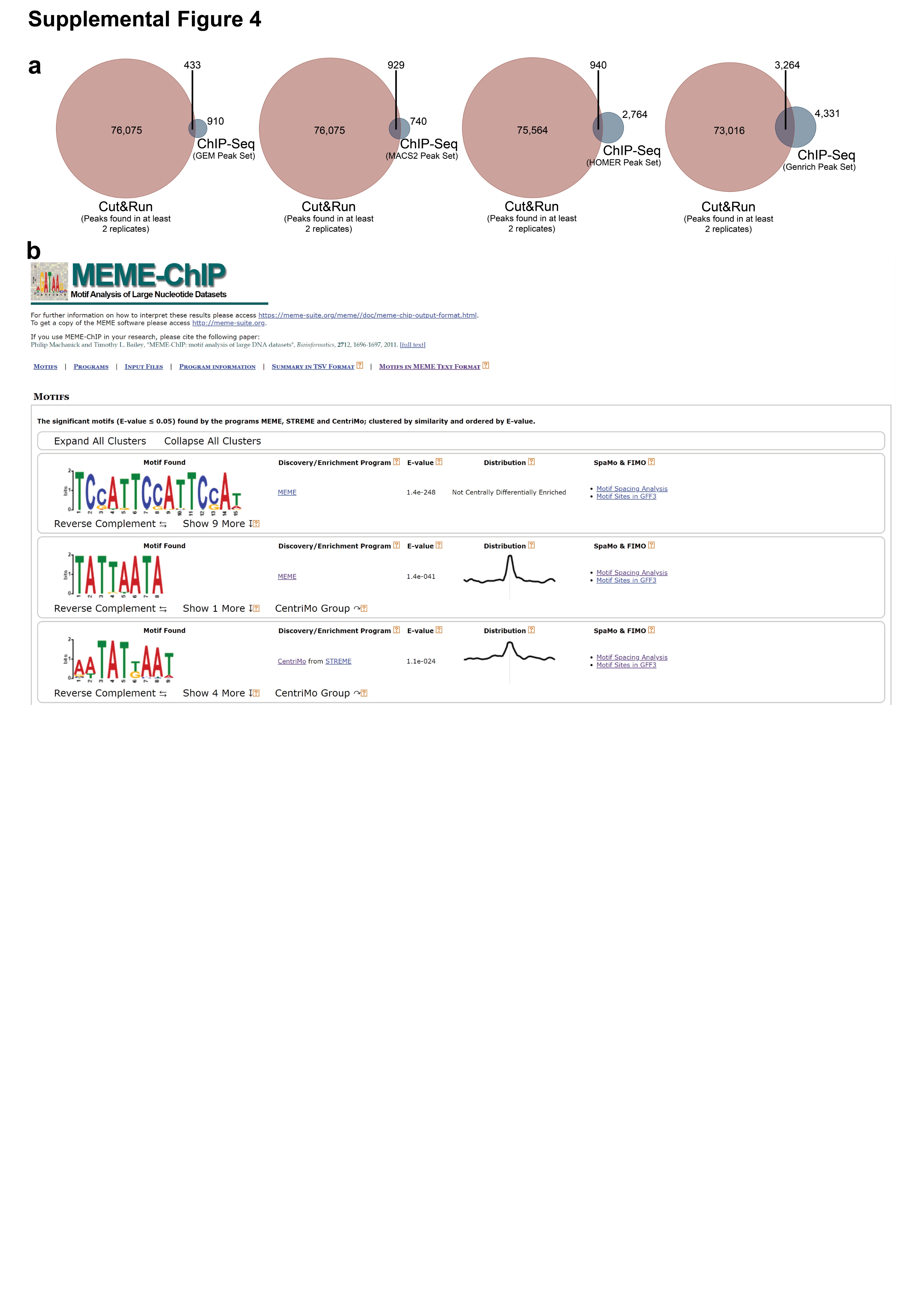

Supplement: Supp_figure4_capturing_lost_peaks_Large_bbab537 [file supp_figure4_capturing_lost_peaks_large_bbab537.jpeg]

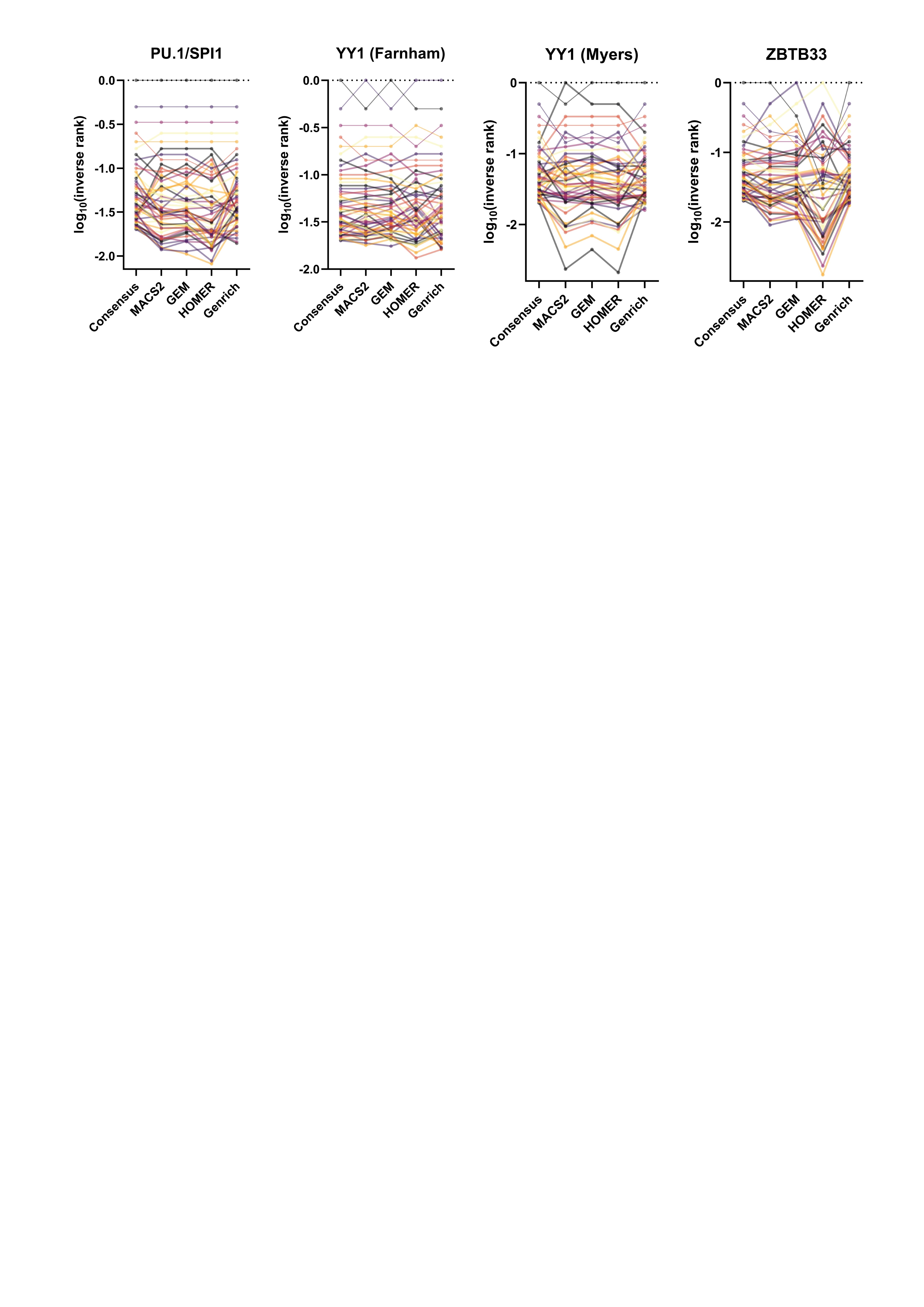

Supplement: Supp_figure2_cosnensus_peaks5_(Large)_bbab537 [file supp_figure2_cosnensus_peaks5_(large)_bbab537.jpeg]
