# Supplementary material for: ChIP-AP: an integrated analysis pipeline for unbiased ChIP-seq analysis
Source: Brief Bioinform. 2021 Dec 30;23(1):bbab537. doi: 10.1093/bib/bbab537 (PMC8769893; doi:10.1093/bib/bbab537)
Supplement: SuppTable2_bbab537 [file supptable2_bbab537.docx]

**Supplemental Table 2**. Motif position bias z-test statistics comparing the consensus peak set to individual peak callers for all profiled ENCODE TFs

| Transcription Factor | Consensus vs GEM p-value | Consensus vs GEM 99% CI | Consensus vs MACS2 p-value | Consensus vs MACS2 99% CI | Consensus vs HOMER p-value | Consensus vs HOMER 99% CI | Consensus vs Genrich p-value | Consensus vs Genrich 99% CI |
| --- | --- | --- | --- | --- | --- | --- | --- | --- |
| ATF4 | < 2.2 * 10^-16^ | -9.12 : -5.25 | 1.75 * 10^-5^ | -5.63 : -1.41 | 7.77 * 10^-9^ | -6.74 : -2.58 | 0.67 | -2.75 : 1.98 |
| CEBPB | < 2.2 * 10^-16^ | -4.14 : -2.79 | < 2.2 * 10^-16^ | -4.40 : -3.08 | < 2.2 * 10^-16^ | -5.20 : -3.95 | 0.87 | -0.80 : 0.91 |
| GATA1 | < 2.2 * 10^-16^ | -11.92 : -6.87 | < 2.2 * 10^-16^ | -13.27 : -8.32 | < 2.2 * 10^-16^ | -14:78 : -9.89 | 0.94 | -3.39 : 3.18 |
| JUN | < 2.2 * 10^-16^ | -10.48 : -5.74 | < 2.2 * 10^-16^ | -11.00 : -6.28 | < 2.2 * 10^-16^ | -11.93 : -7.26 | 0.96 | -3.17 : 3.05 |
| MAFF | 3.81 * 10^-9^ | -4.42 : -1.73 | 1.15 * 10^-11^ | -4.79 : -2.15 | 1.29 * 10^-13^ | -5.11 : -2.47 | 0.97 | -1.66 : 1.71 |
| MAX | 0.018 | -16.75 : 0.72 | 0.95 | -10.24 : 9.71 | 0.10 | -14.95 : 3.28 | 0.74 | -11.17 : 8.56 |
| MEIS2 | 6.33 * 10^-5^ | -7.95 : -1.72 | 1.17 * 10^-5^ | -8.20 : -2.13 | 1.78 * 10^-6^ | -8.82 : -2.64 | 0.52 | -2.75 : 4.56 |
| RUNX1 | 1.22 * 10^-5^ | -26.90 : -6.96 | 1.13 * 10^-3^ | -22.87 : -2.66 | 8.69 * 10^-9^ | -31.17 : -11.89 | 0.43 | -17.95 : 9.56 |
| SPI1 | < 2.2 * 10^-16^ | -10.90 : -7.96 | < 2.2 * 10^-16^ | -24.82 : -15.90 | < 2.2 * 10^-16^ | -24.82 : -15.19 | 0.51 | -24.82 : -24.37 |
| ZBTB33 | 0.55 | -10.50 : 6.52 | 0.41 | -11.11 : 5.70 | 0.14 | -12.42 : 3.44 | 0.88 | -9.50 : 8.41 |
