# Supplementary material for: ChIP-AP: an integrated analysis pipeline for unbiased ChIP-seq analysis
Source: Brief Bioinform. 2021 Dec 30;23(1):bbab537. doi: 10.1093/bib/bbab537 (PMC8769893; doi:10.1093/bib/bbab537)
Supplement: SuppTable5_bbab537 [file supptable5_bbab537.docx]

**Supplemental Table 5**. Top 20 RUNX1 GO terms for the Consensus Peak Set and the MACS2 Peak Set

| Consensus Peak Set Gene Ontology (Biological Processes (Results) | | | | MACS2 Peak Set Gene Ontology (Biological Processes (Results) | | | |
| --- | --- | --- | --- | --- | --- | --- | --- |
| TermID | **Term** | **Enrichment** | **Target Genes  in Term** | **TermID** | **Term** | **Enrichment** | **Target Genes  in Term** |
| GO:0065009 | regulation of molecular function | 1.914*10^06^ | 81 | GO:0048518 | positive regulation of biological process | 6.4519*10^13^ | 857 |
| GO:0010506 | regulation of autophagy | 3.525*10^05^ | 17 | GO:0043412 | macromolecule modification | 7.94864*10^13^ | 494 |
| GO:1903146 | regulation of autophagy of mitochondrion | 5.875*10^05^ | 6 | GO:0031323 | regulation of cellular metabolic process | 9.50085*10^13^ | 866 |
| GO:2000973 | regulation of pro-B cell differentiation | 0.000145 | 3 | GO:0031325 | positive regulation of cellular metabolic process | 1.02492*10^12^ | 500 |
| GO:1900221 | regulation of amyloid-beta clearance | 0.000180 | 4 | GO:0036211 | protein modification process | 1.27631*10^12^ | 466 |
| GO:1905456 | regulation of lymphoid progenitor cell differentiation | 0.000229 | 3 | GO:0006464 | cellular protein modification process | 1.27631*10^12^ | 466 |
| GO:0050790 | regulation of catalytic activity | 0.000232 | 60 | GO:0009893 | positive regulation of metabolic process | 1.76969*10^12^ | 539 |
| GO:0043085 | positive regulation of catalytic activity | 0.000269 | 41 | GO:0048522 | positive regulation of cellular process | 2.30811*10^12^ | 761 |
| GO:1903432 | regulation of TORC1 signaling | 0.000324 | 5 | GO:0048523 | negative regulation of cellular process | 2.61013*10^12^ | 687 |
| GO:0044093 | positive regulation of molecular function | 0.000335 | 48 | GO:0010604 | positive regulation of macromolecule metabolic process | 2.61895*10^12^ | 503 |
| GO:2000169 | regulation of peptidyl-cysteine S-nitrosylation | 0.000340 | 3 | GO:0051173 | positive regulation of nitrogen compound metabolic process | 9.01509*10^12^ | 476 |
| GO:0032870 | cellular response to hormone stimulus | 0.000405 | 22 | GO:0050790 | regulation of catalytic activity | 1.33121*10^11^ | 366 |
| GO:1900222 | negative regulation of amyloid-beta clearance | 0.000480 | 3 | GO:0065009 | regulation of molecular function | 1.43638*10^11^ | 452 |
| GO:0016241 | regulation of macroautophagy | 0.000532 | 10 | GO:0044260 | cellular macromolecule metabolic process | 1.77626*10^11^ | 705 |
| GO:0070887 | cellular response to chemical stimulus | 0.000588 | 69 | GO:0071840 | cellular component organization or biogenesis | 2.52301*10^11^ | 801 |
| GO:1901701 | cellular response to oxygen-containing compound | 0.000637 | 31 | GO:0016043 | cellular component organization | 5.19017*10^11^ | 776 |
| GO:0010821 | regulation of mitochondrion organization | 0.000759 | 10 | GO:0051128 | regulation of cellular component organization | 9.09758*10^11^ | 380 |
| GO:0006391 | transcription initiation from mitochondrial promoter | 0.000794 | 2 | GO:1901564 | organonitrogen compound metabolic process | 1.39081*10^10^ | 726 |
| GO:0051716 | cellular response to stimulus | 0.000920 | 135 | GO:0033043 | regulation of organelle organization | 2.98496*10^10^ | 219 |
